# Fractal memory structure in the spatiotemporal learning rule

**DOI:** 10.3389/fncom.2025.1641519

**Published:** 2025-12-16

**Authors:** Takemori Orima, Ichiro Tsuda, Minoru Tsukada, Hiromichi Tsukada, Yoshihiko Horio

**Affiliations:** 1 Advanced Comprehensive Research Organization, Teikyo University, Itabashi, Japan; 2 AIT Center, Sapporo City University, Sapporo, Japan; 3 Brain Science Institute, Tamagawa University, Machida, Japan; 4 Center for Mathematical Science and Artificial Intelligence, Chubu University, Kasugai, Japan

**Keywords:** fractal structure, spatiotemporal learning rule, hippocampus (CA1), neural network, one-shot learning

## Abstract

The spatiotemporal learning rule (STLR) can reproduce synaptic plasticity in the hippocampus. Analysis of the synaptic weights in the network with the STLR is challenging. Consequently, our previous research only focused on the network's outputs. However, a detailed analysis of the STLR requires focusing on the synaptic weights themselves. To address this issue, we mapped the synaptic weights to a distance space and analyzed the characteristics of the STLR. The results indicate that the synaptic weights form a fractal-like structure in Euclidean distance space. Furthermore, three analytical approaches—multi-dimensional scaling, estimating fractal dimension, and modeling with an iterated function system—demonstrate that the STLR forms a fractal structure in the synaptic weights through fractal coding. These findings contribute to clarifying the learning mechanisms in the hippocampus.

## 1 Introduction

In the brain, the hippocampus plays a crucial role in forming episodic memories by storing spatiotemporal contexts (Kovcs, 2020). This process requires real-time, online learning, where patterns must be processed sequentially as they arrive, rather than being stored in a buffer for offline batch training (Melchior et al., 2024). However, current artificial neural networks often suffer from catastrophic forgetting, making online learning significantly more challenging than offline learning (McCloskey and Cohen, 1989). One reason for this limitation is their common reliance on Hebbian-type learning rule (Hebb, 1949). Hebbian-type learning, based on classical conditioning, updates synaptic weights according to the correlation between inputs and outputs. In contrast, the spatiotemporal learning rule (STLR) (Tsukada et al., 1996), discovered through physiological experiments in the hippocampus, differs fundamentally from Hebbian-type learning. The STLR updates synaptic weights based on correlations among inputs.

The STLR was proposed to model the synaptic plasticity in the CA1 region (Tsukada et al., 1996) as a specific learning rule for the hippocampal neurons. The CA1 region, as the output area of the hippocampus, encodes inputs from CA3 (Guardamagna et al., 2023) to set up associatively learned backprojections to the neocortex, allowing subsequent retrieval of information to the neocortex (Rolls, 2010). In this paper, the single-layer feedforward neural network with STLR synapses models this CA1 region.

Furthermore, it has been reported that networks utilizing the STLR demonstrate superior separation performance in tasks that are difficult for Hebbian learning rules (Tsukada and Pan, 2005). In this network, the synaptic weight values are updated using the coincidence among the inputs to the neuron connected through the STLR synapses. The network learns slight differences in similar spatiotemporal patterns inputted from CA3 to CA1. These spatiotemporal patterns are time series consisting of binary spatial vectors with small Hamming distances (Tsukada and Tsukada, 2021a). After learning them, a histogram of the synaptic weight values shows a multimodal distribution reflecting the temporal order of the input spatial vectors (Tsukada and Tsukada, 2021a). In other words, the network learned slight differences in the temporal order of the constituent spatial patterns of the spatiotemporal inputs, which is not possible with the Hebbian learning rule. A histogram of the Hamming distances between the outputs also shows a multimodal distribution obtained by applying a specific readout pattern to the network (Tsuji et al., 2023a). Additionally, an order-nested structure is observed in the output space using a two-dimensional distance map created according to the temporal order of the input spatial vectors, and this structure has been analyzed in detail in our previous study (Tsuji et al., 2023a). The order-nested structure in the output space strongly suggests that the STLR formed a fractal structure in the synaptic weight space.

A fractal structure in the CA1 region by fractal coding has been previously discussed in the literature (Fukushima et al., 2007). The hippocampus uses fractal properties such as self-similarity and scale-invariance (Husain et al., 2022) to facilitate efficient information compression and processing (Bieberich, 2002; Werner, 2010; Tsuda, 2001; Kuroda et al., 2009). For example, fractal coding embeds high-dimensional spatiotemporal information into a low-dimensional synaptic weight space. Consequently, the hippocampus can efficiently use limited resources, such as the number of neurons and synaptic connections. This feature is also useful for engineering applications, such as edge devices (Orima et al., 2023). One example of fractal coding is a contraction-mapping system (Tsuda, 2001), which memorizes information as an attractor, driven by chaotic signals. Another example is a feedforward neural network (Yamaguti et al., 2011), which learns and recalls patterns generated by a Cantor set using Hebbian learning rule (Hebb, 1949) synapses. However, in these models, learning itself was not the primary mechanism for generating the fractal structure.

In this study, we confirm that the STLR can form a fractal structure solely through learning. Furthermore, we demonstrated that the order-nested structure in the output space is a reliable and practical indicator of the fractal structure in the synaptic weight space. The following three analysis methods were employed, as it was difficult to theoretically prove the formation of a fractal structure: The first method involved multi-dimensional scaling (MDS) (Torgerson, 1952) to qualitatively confirm the fractal structure in the synaptic weight space. Second, the box-counting (Mandelbrot, 1982; Sarkar and Chaudhuri, 1994), lacunarity (Mandelbrot, 1994; Smith et al., 1996), and mass-radius (Landini and Rippin, 1993) methods were used to quantitatively evaluate the fractal dimension and structure in the synaptic weight space. Third, modeling with an iterated function system (IFS) was employed for mathematical analysis. The following section briefly describes the STLR, neurons, network configuration, and initialization processes used in this study.

## 2 Materials and methods

### 2.1 Synapse with STLR

**Figure 1:**
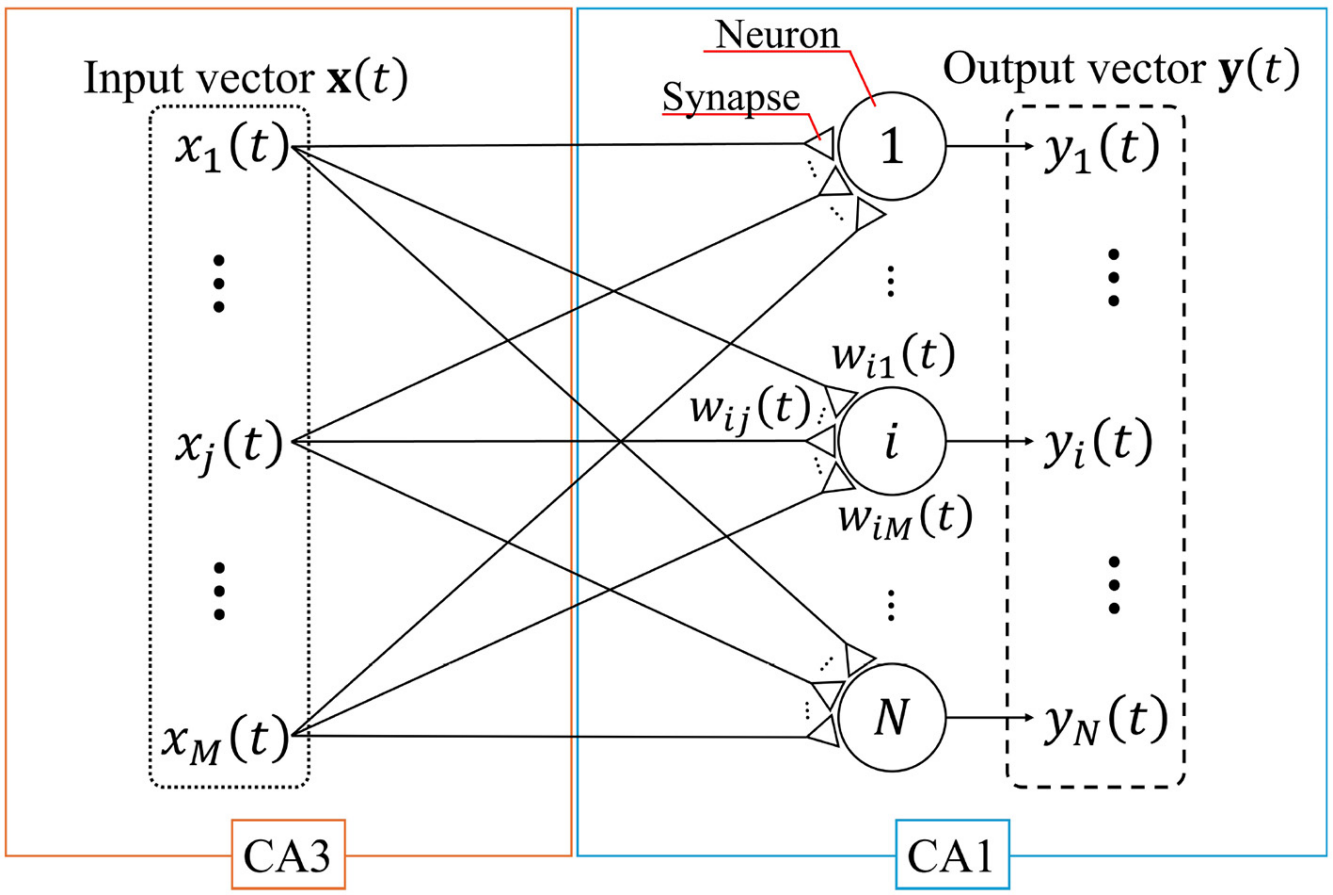
Single-layer feedforward neural network. This network models a connection from CA3 to CA1. The large circles and small triangles represent neurons and synapses, respectively. Synapses are updated based on the STLR. The input vector **x**(*t*) and output vector **y**(*t*) consist of *M* elements (enclosed by the dotted line) and *N* elements (enclosed by the dashed line), respectively.

A single-layer feedforward neural network with the STLR synapses that models the connection from CA3 to CA1 (Tsukada and Pan, 2005) is shown in Figure 1. In this figure, large circles and small triangles represent neurons and synapses, respectively; **x**(*t*) and **y**(*t*) are the input and output column vectors of the network at discrete time *t*, respectively; *x*_*j*_(*t*) and *y*_*i*_(*t*) are the *j*th element of **x**(*t*) and *i*th element of **y**(*t*), respectively; *M* and *N* are the number of inputs and neurons, respectively; and *w*_*ij*_(*t*) is the synaptic weight connected from the *j*th input to the *i*th neuron.

The value of *w*_*ij*_(*t*) is updated based on the STLR (Tsukada et al., 1996) using the following equations:


$$\begin{array}{l} w_{ij}\left(t+1\right)=w_{ij}\left(t\right)+\Delta w_{ij}\left(t\right), \end{array}$$ (1)

$$\Delta w_{ij}(t)=\{\begin{array}{ll} \eta & (q_{ij}(t)>\theta_{\text{LTP}}),\\ 0 & (\theta_{\text{LTD}}\leq q_{ij}(t)\leq \theta_{\text{LTP}}),\\ -\eta & (q_{ij}(t)<\theta_{\text{LTD}}), \end{array}$$ (2)

where η, θ_LTP_, θ_LTD_, and *q*_*ij*_(*t*) are the learning constant, long-term potentiation (LTP) threshold, long-term depression (LTD) threshold, and time history of the coincidence among the inputs to the *i*th neuron from the *j*th synapse (hereafter referred to as the input coincidence), respectively. The time history *q*_*ij*_(*t*) is defined as follows:


$$\begin{array}{l} q_{ij}\left(t\right)=\overset{t}{\underset{t^{\prime }=0}{\sum }}c_{ij}\left(t^{\prime }\right)\exp\left(-\frac{t-t^{\prime }}{\tau_{\text{Q}}}\right), \end{array}$$ (3)

$$\begin{array}{l} c_{ij}\left(t\right)=w_{ij}\left(t\right)x_{j}\left(t\right)\overset{M}{\underset{j^{\prime }=1,j^{\prime }\neq j}{\sum }}w_{ij^{\prime }}\left(t\right)x_{j^{\prime }}\left(t\right), \end{array}$$

$$\begin{array}{ll} \;= & w_{ij}\left(t\right)x_{j}\left(t\right)\left(p_{i}\left(t\right)-w_{ij}\left(t\right)x_{j}\left(t\right)\right), \end{array}$$ (4)

where τ_Q_, *p*_*i*_(*t*), and *c*_*ij*_(*t*) are the time constants, the internal state of the *i*th neuron, and the input coincidence of the *ij*th synapse, respectively. The network learns the spatial and temporal structures of the input spatiotemporal patterns through *c*_*ij*_(*t*) and *q*_*ij*_(*t*), respectively.

### 2.2 Neuron

For simplicity, we employ an analog neuron model in the network shown in Figure 1 (Tsukada and Tsukada, 2021a). The internal state and output of the *i*th neuron in the network are expressed as


$$\begin{array}{l} p_{i}\left(t\right)=\overset{M}{\underset{j=1}{\sum }}w_{ij}\left(t\right)x_{j}\left(t\right), \end{array}$$ (5)

$$y_{i}(t)=f\left(p_{i}(t)\right)=\{\begin{array}{ll} 1 & (p_{i}(t)\geq \theta ),\\ 0 & (p_{i}(t)<\theta ), \end{array}$$ (6)

where *p*_*i*_(*t*) and *y*_*i*_(*t*) denote the internal state and output of the *i*th neuron, respectively; *f*(·) is the output function of the neuron (step function); and θ is the firing threshold of the neuron.

### 2.3 Network configuration

The network shown in Figure 1 consists of the synapses and neurons, as described in Sections 2.1 and 2.2. The network learns the input spatiotemporal patterns that are slightly different (Section 2.4). We initialize the synaptic weights to the same initial values for all of the input spatiotemporal patterns. After learning each spatiotemporal pattern, the synaptic weights of the network are fixed, and a common readout pattern is input (Section 2.5).

In the following sections, we describe the procedure for generating spatiotemporal patterns and the details of the numerical simulation experiments.

### 2.4 Input spatiotemporal patterns

Input spatiotemporal patterns, each composed of a time series of binary spatial vectors with small Hamming distances from one another, are used to facilitate detailed analyses. The procedure for generating a spatiotemporal pattern **X** is as follows:


$$\begin{array}{l} \text{X}=\left(\text{x}\left(1\right),\cdots \;,\text{x}\left(t\right),\cdots \;,\text{x}\left(T\right)\right), \end{array}$$ (7)

$$\begin{array}{l} \text{x}\left(t\right)={\left[x_{1}\left(t\right),\cdots \;,x_{j}\left(t\right),\cdots \;,x_{M}\left(t\right)\right]}^{⊤}, \end{array}$$ (8)

where (·) and [·] denote a vector time-series and vector, respectively, ⊤ denotes transposition, and *T* is the length of **X** (in this study, *T* = 5).

At time *t*, one of the spatial vectors is input as


$$\begin{array}{l} \text{x}\left(t\right)\in \left\{{\text{z}}^{1},\cdots \;,{\text{z}}^{k},\cdots \;,{\text{z}}^{K}\right\}, \end{array}$$ (9)

where {·}, *K*, **z**^*k*^, and *k* denote a set, the number of spatial vectors, the spatial vector, and its index of the spatial vector, respectively. In the present study, the three different spatial vectors (*K* = 3) are randomly generated under the following constraints:


$$\begin{array}{l} {\text{z}}^{k}={\left[z_{1}^{k},\cdots \;,z_{j}^{k},\cdots \;,z_{M}^{k}\right]}^{⊤},\;1\leq k\leq 3, \end{array}$$ (10)

$$\begin{array}{l} z_{j}^{k}\in \left\{0,1\right\},\overset{M}{\underset{j=1}{\sum }}z_{j}^{k}=\frac{M}{2}, \end{array}$$ (11)

$$\begin{array}{l} {\text{z}}^{1}\neq {\text{z}}^{2},{\text{z}}^{2}\neq {\text{z}}^{3},{\text{z}}^{3}\neq {\text{z}}^{1}. \end{array}$$ (12)

Some elements of **z**^*k*^ are bit-flipped such that the Hamming distance between them is 10 (Tsukada and Tsukada, 2021a).


$$\begin{array}{l} HD\left({\text{z}}^{1},{\text{z}}^{2}\right)=HD\left({\text{z}}^{2},{\text{z}}^{3}\right)=HD\left({\text{z}}^{3},{\text{z}}^{1}\right)=10, \end{array}$$ (13)

where *HD*(**x**, **y**) is the Hamming distance between the vectors **x** and **y**. The total number *L* of spatiotemporal patterns is *K*^*T*^ = 3^5^ = 243, which are generated using **z**^1^, **z**^2^, and **z**^3^ as follows:


$$\begin{array}{l} X\in \{(z^{1},z^{1},\cdots ,z^{1}),(z^{1},z^{1},\cdots ,z^{2}),\cdots ,(z^{3},z^{3},\cdots ,z^{2}),\\ \;\left(z^{3},z^{3},\cdots ,z^{3}\right)\}. \end{array}$$ (14)

Finally, the generated spatiotemporal patterns are labeled using *k* and arranged in increasing order. The labels of the spatiotemporal patterns are shown in Table 1. In this table, $k_{t}^{l}$ denotes the index of the spatial vector at *t* in the *l*th spatiotemporal pattern.

**Table 1:** Labels of the spatiotemporal patterns.

**Label**	**Spatiotemporal pattern**
1	(**z**^1^, **z**^1^, ⋯ , **z**^1^)
2	(**z**^1^, **z**^1^, ⋯ , **z**^2^)
⋮	⋮
*l*	$\left({\text{z}}^{k_{1}^{l}},\cdots \;,{\text{z}}^{k_{t}^{l}},\cdots \;,{\text{z}}^{k_{5}^{l}}\right)$
⋮	⋮
242	(**z**^3^, **z**^3^, ⋯ , **z**^2^)
243	(**z**^3^, **z**^3^, ⋯ , **z**^3^)

**Figure 2:**
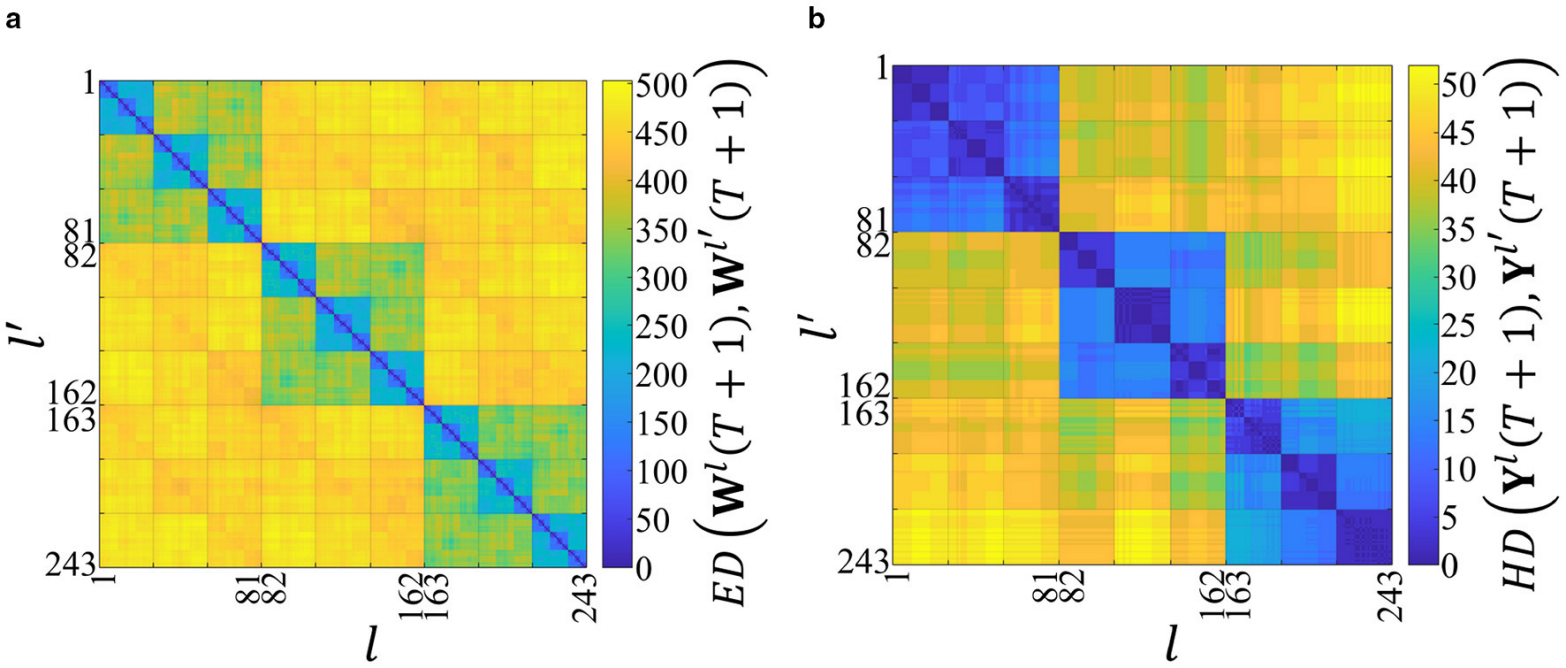
Structures in the distance space of the synaptic weights and the outputs after the network has learned 243 spatiotemporal patterns. The horizontal and vertical axes represent the labels of the input spatiotemporal patterns, and the color bars represent the distances. **(a)** Two-dimensional distance map using D_w_(*T*+1). The color of each point (*l, l*′) represents the Euclidean distance *ED*(W^*l*^(*T*+1), W^*l*^′(*T*+1)) between the final weight matrices learned from patterns *l* and *l*′. The color scale indicates that warmer colors (yellow) represent greater distances. The distinct squares along the diagonal reveal a clear hierarchical and self-similar clustering, which we identify as a fractal-like structure. **(b)** Two-dimensional distance map using D_y_(*T*+1). The color represents the Hamming distance *HD*(Y^*l*^(*T*+1), Y^*l*^′(*T*+1)) between the corresponding output vectors. This map exhibits a similar hierarchical clustering, which we term an order-nested structure, reflecting the temporal order of the input patterns. The high structural similarity between **(a)** and **(b)** suggests that the output space reflects the fractal-like structure of the synaptic weight space.

Each pattern consists of a sequence of three spatial vectors. The notation $k_{t}^{l}$ represents the index (1, 2, or 3) of the spatial vector **z**^*k*^ at a time *t* in the *l*th spatiotemporal pattern. The patterns are labeled in increasing order. These labels correspond to the arrangement of the horizontal and vertical axes in the distance maps shown in Figure 2.

### 2.5 Numerical simulation experiment

A numerical simulation experiment, consisting of a learning phase, a reading phase, and an evaluation, is subsequently conducted.

First, for the learning phase, we set the initial values of the network and define a synaptic weight matrix. Additionally, we conduct experiments where white noise is applied to the input. The amplitude of the white noise is based on the standard deviation of the internal state of a neuron in the noise-free condition (for detailed statistics, such as the standard deviation of the internal state without noise, see Supplementary Section 1).

Second, for the reading phase, we define an output vector obtained by inputting the readout signal. The firing threshold of neurons is adjusted so that information embedded in the synaptic weights is exposed in the output.

Finally, for the evaluation, we create two-dimensional distance matrices using synaptic weight matrices and output vectors obtained in the learning and reading phases. To investigate the relationship between the synaptic weight and output structures, we calculate the similarity between the two-dimensional distance matrices. We further confirm the fractal structure of the synaptic weights using the MDS method and estimate the fractal dimensions using the box-counting, lacunarity, and mass-radius methods. We also analyzed the synaptic weight structure using Isomap (Tenenbaum et al., 2000) and UMAP (McInnes et al., 2020), which are common dimensionality reduction methods (for detailed results, see Supplementary Section 6). Additionally, based on the results of the MDS analysis, we mathematically demonstrate that a fractal structure is formed in the synaptic weight space through modeling with an IFS.

#### 2.5.1 Learning phase

The network learns the *L* spatiotemporal patterns generated in Section 2.4. First, the initial values of the synaptic weights are set as follows:


$$\begin{array}{l} w_{ij}\left(0\right)\sim U\left(-1,1\right), \end{array}$$ (15)

where *U*(*a, b*) denotes a uniform random number ranging from *a* to *b*. The other variables are initialized to zero, as Δ*w*_*ij*_(0) = *q*_*ij*_(0) = *c*_*ij*_(0) = *x*_*j*_(0) = *p*_*i*_(0) = *y*_*i*_(0) = 0.

Second, the network learns one *l*th of the 243 spatiotemporal patterns. At *t* = 1, $\text{x}\left(1\right)={\text{z}}^{k_{1}^{l}}$ is the input to the network, and the states of the neurons are updated using Equations 5 and 6. The synaptic weight values are then updated using Equations 1–4. Similarly, ${\text{z}}^{k_{2}^{l}},{\text{z}}^{k_{3}^{l}},\cdots \;,{\text{z}}^{k_{T}^{l}}$ are input to the network at *t* = 2, 3, ⋯ , *T* and the neuron states and synaptic weight values are updated at each time. At *t* = *T*+1, the update of the synaptic weight values is stopped and the synaptic weight matrix is defined as


$$W^{l}(T+1)=\left[\begin{array}{ccccc} w_{11}^{l}(T+1) & \cdots & w_{1j}^{l}(T+1) & \cdots & w_{1M}^{l}(T+1)\\ \vdots & \ddots & \; & \; & \vdots \\ w_{i1}^{l}(T+1) & \; & w_{ij}^{l}(T+1) & \; & w_{iM}^{l}(T+1)\\ \vdots & \; & \; & \ddots & \vdots \\ w_{N1}^{l}(T+1) & \cdots & w_{Nj}^{l}(T+1) & \cdots & w_{NM}^{l}(T+1) \end{array}\right].$$ (16)

Numerical simulations are also performed when noise is applied to the input. In such cases, the noise amplitude is varied relative to the standard deviation of the internal state σ_*p*_ at *t* = 1 under noise-free conditions. For details on statistics of the network at *t* = 1, such as σ_*p*_, see Supplementary Section 1.

#### 2.5.2 Reading phase

To read out the information memorized in the synaptic weights, the readout pattern **z**^read^ is input to the network with fixed synaptic weights, where **z**^read^ satisfies the following equation:


$$\begin{array}{lll} {\text{z}}^{\text{read}} & \neq & {\text{z}}^{1},{\text{z}}^{\text{read}}\neq {\text{z}}^{2},{\text{z}}^{\text{read}}\neq {\text{z}}^{3}, \end{array}$$ (17)

$$\begin{array}{l} HD\left({\text{z}}^{\text{read}},{\text{z}}^{1}\right)=HD\left({\text{z}}^{\text{read}},{\text{z}}^{2}\right)=HD\left({\text{z}}^{\text{read}},{\text{z}}^{3}\right)=10. \end{array}$$ (18)

The synaptic weight values of the network are fixed to **W**^*l*^(*T*+1), **z**^read^ is input to the network (e.g., **x**(*T*+1) = **z**^read^), and the states of the neurons are updated using Equations 5 and 6.

The output vector is obtained as follows:


$$\begin{array}{l} {\text{Y}}^{l}\left(T+1\right)={\left[y_{1}^{l}\left(T+1\right),\cdots \;,y_{i}^{l}\left(T+1\right),\cdots \;,y_{N}^{l}\left(T+1\right)\right]}^{⊤}, \end{array}$$ (19)

where θ in Equation 6 is adjusted to ensure that the *N*/2 neurons are fired.

#### 2.5.3 Evaluation

First, we investigate the relationship between **W**^*l*^(*t*) and **Y**^*l*^(*t*) in distance space. To analyze the structure of **W**^*l*^(*t*), the Euclidean distances between the synaptic weight matrices are calculated using the following equation:


$$\begin{array}{l} ED\left({\text{W}}^{l}\left(t\right),{\text{W}}^{l^{\prime }}\left(t\right)\right)=\sqrt{\overset{N}{\underset{i=1}{\sum }}\overset{M}{\underset{j=1}{\sum }}{\left(w_{ij}^{l}\left(t\right)-w_{ij}^{l^{\prime }}\left(t\right)\right)}^{2}}. \end{array}$$ (20)

The distance matrix in the synaptic weight space **D**_w_(*t*) is defined using *ED*(**W**^*l*^(*t*), **W**^*l*^′(*t*)) by arranging the rows and columns in increasing order using *l* as follows:


$$D_{\text{w}}(t)=\left[\begin{array}{ccccc} ED\left(W^{1}(t),W^{1}(t)\right) & \cdots & ED\left(W^{1}(t),W^{l^{\prime }}(t)\right) & \cdots & ED\left(W^{1}(t),W^{L}(t)\right)\\ \vdots & \ddots & \; & \; & \vdots \\ ED\left(W^{l}(t),W^{1}(t)\right) & \; & ED\left(W^{l}(t),W^{l^{\prime }}(t)\right) & \; & ED\left(W^{l}(t),W^{L}(t)\right)\\ \vdots & \; & \; & \ddots & \vdots \\ ED\left(W^{L}(t),W^{l}(t)\right) & \cdots & ED\left(W^{L}(t),W^{l^{\prime }}(t)\right) & \cdots & ED\left(W^{L}(t),W^{L}(t)\right) \end{array}\right].$$ (21)

In contrast, the distance matrix in the output space **D**_y_(*t*) obtained from the Hamming distance *HD*(**Y**^*l*^(*t*), **Y**^*l*^′(*t*)) between the output vectors is defined by


$$D_{\text{y}}(t)=\left[\begin{array}{ccccc} HD\left(Y^{1}(t),Y^{1}(t)\right) & \cdots & HD\left(Y^{1}(t),Y^{l^{\prime }}(t)\right) & \cdots & HD\left(Y^{1}(t),Y^{L}(t)\right)\\ \vdots & \ddots & \; & \; & \vdots \\ HD\left(Y^{l}(t),Y^{1}(t)\right) & \; & HD\left(Y^{l}(t),Y^{l^{\prime }}(t)\right) & \; & HD\left(Y^{l}(t),Y^{L}(t)\right)\\ \vdots & \; & \; & \ddots & \vdots \\ HD\left(Y^{L}(t),Y^{1}(t)\right) & \cdots & HD\left(Y^{L}(t),Y^{l^{\prime }}(t)\right) & \cdots & HD\left(Y^{L}(t),Y^{L}(t)\right) \end{array}\right].$$ (22)

We further create two-dimensional distance maps using **D**_w_(*t*) and **D**_y_(*t*) and qualitatively confirm the structural similarity between the synaptic weight space and the output space. Furthermore, the cosine similarity is calculated to quantitatively analyze the structural similarity between **D**_w_(*t*) and **D**_y_(*t*) as follows:


$$\begin{array}{l} CS\left(t\right)=\frac{{\text{D}}_{\text{w}}\left(t\right)\cdot {\text{D}}_{\text{y}}\left(t\right)}{||{\text{D}}_{\text{w}}\left(t\right)||||{\text{D}}_{\text{y}}\left(t\right)||}, \end{array}$$ (23)

where · and ||**D**|| are the sum of the Hadamard product and Frobenius norm of matrix **D**, respectively, and are calculated as


$$\begin{array}{l} {\text{D}}_{\text{w}}\left(t\right)\cdot {\text{D}}_{\text{y}}\left(t\right)=\overset{L}{\underset{l=1}{\sum }}\overset{L}{\underset{l^{\prime }=1}{\sum }}ED\left({\text{W}}^{l}\left(t\right),{\text{W}}^{l^{\prime }}\left(t\right)\right) \end{array}$$

$$\begin{array}{l} HD\left({\text{Y}}^{l}\left(t\right),{\text{Y}}^{l^{\prime }}\left(t\right)\right), \end{array}$$ (24)

$$\begin{array}{l} ||{\text{D}}_{\text{w}}\left(t\right)||=\sqrt{\overset{L}{\underset{l=1}{\sum }}\overset{L}{\underset{l^{\prime }=1}{\sum }}ED{\left({\text{W}}^{l}\left(t\right),{\text{W}}^{l^{\prime }}\left(t\right)\right)}^{2}}, \end{array}$$ (25)

$$\begin{array}{l} ||{\text{D}}_{\text{y}}\left(t\right)||=\sqrt{\overset{L}{\underset{l=1}{\sum }}\overset{L}{\underset{l^{\prime }=1}{\sum }}HD{\left({\text{Y}}^{l}\left(t\right),{\text{Y}}^{l^{\prime }}\left(t\right)\right)}^{2}}. \end{array}$$ (26)

Second, the MDS method is used to clearly visualize the structure of **D**_w_(*t*) as it is an appropriate method for expressing the distances between the synaptic weight matrices in low dimensions. A reasonable compressed dimension *n* (1 ≤ *n* ≤ *L*) is estimated to reduce the dimensions to a lower-dimensional space without losing as much high-dimensional information as **D**_w_(*t*). The dimension-compressed distance matrix in the synaptic weight space **G**_w_(*t*) is defined as


$$\begin{array}{l} {\text{G}}_{\text{w}}\left(t\right)={\left[{\text{g}}^{1}\left(t\right),\cdots \;,{\text{g}}^{l}\left(t\right),\cdots \;,{\text{g}}^{L}\left(t\right)\right]}^{⊤}\in {\mathbb{R}}^{L\times n}, \end{array}$$ (27)

$$\begin{array}{l} {\text{g}}^{l}\left(t\right)=\left[g_{1}^{l}\left(t\right),\cdots \;,g_{m}^{l}\left(t\right),\cdots \;,g_{n}^{l}\left(t\right)\right]\in {\mathbb{R}}^{n}, \end{array}$$ (28)

where **g**^*l*^(*t*) (1 ≤ *l* ≤ *L*) is a coordinate in the distance space compressed into *n*-dimensions, and $g_{m}^{l}\left(t\right)$ is the *m*th element in **g**^*l*^(*t*). To estimate *n*, double centering is used to calculate a symmetric matrix ${\text{G}}_{\text{w}}\left(t\right){\text{G}}_{\text{w}}{\left(t\right)}^{⊤}$ as follows:


$$\begin{array}{l} {\text{G}}_{\text{w}}\left(t\right){\text{G}}_{\text{w}}{\left(t\right)}^{⊤}=-\frac{1}{2}\left(\text{I}-\frac{1}{L}\text{J}\right){\text{D}}_{\text{w}}\left(t\right)\left(\text{I}-\frac{1}{L}\text{J}\right), \end{array}$$ (29)

where **I** and **J** ∈ℝ^*L*×*L*^ are defined as


$$\begin{array}{l} \text{I}=\left[\begin{array}{cccc} 1 & 0 & \cdots & 0\\ 0 & 1 & \cdots & 0\\ \vdots & \vdots & \ddots & \vdots \\ 0 & 0 & \cdots & 1 \end{array}\right],\text{J}=\left[\begin{array}{cccc} 1 & 1 & \cdots & 1\\ 1 & 1 & \cdots & 1\\ \vdots & \vdots & \ddots & \vdots \\ 1 & 1 & \cdots & 1 \end{array}\right]. \end{array}$$ (30)

The right-hand side of Equation 29 contains *L* eigenvalues. The sum of *L* eigenvalues $\Lambda_{L}=\overset{L}{\underset{m=1}{\sum }}\lambda_{m}$ is used as a criterion for estimating *n*, where λ_*m*_ is the *m*th eigenvalue and *m* is an index when the eigenvalues are sorted in descending order. In the left-hand side of Equation 29, the sum of the eigenvalues $\Lambda_{n}=\overset{n}{\underset{m=1}{\sum }}\lambda_{m}$ of ${\text{G}}_{\text{w}}\left(t\right){\text{G}}_{\text{w}}{\left(t\right)}^{⊤}$ is calculated using *n* eigenvectors obtained from the right-hand side of Equation 29. If Λ_*n*_≥0.5Λ_*L*_, *n* is a reasonably compressed dimension. The dimension-compressed position **g**^*l*^(*t*), which is compressed into *n*-dimensions, is labeled *r* according to the temporal order in **X** and is classified into Cluster *r*. By applying the MDS method to each Cluster *r*, the structure within the Cluster *r* was analyzed in greater detail. This operation is repeated *T*−1.

Third, we evaluate the fractal dimension and structure of **G**_w_(*t*) using the box-counting (Mandelbrot, 1982), lacunarity (Mandelbrot, 1994), and mass-radius (Smith et al., 1996) methods. However, it is difficult to apply these methods to **G**_w_(*t*) because **G**_w_(*t*) loses the high-dimensional information of **D**_w_(*t*). Therefore, **G**_w_(*t*) is adjusted to compensate for the lost information of **D**_w_(*t*), and an adjusted distance matrix in the synaptic weight space ${\text{G}}_{\text{w}}^{\text{adj}}\left(t\right)$ is derived. As an adjustment method, the center of the Cluster *r* is defined as *CC*_*r*_ and adjusted using the eigenvalues obtained from Equation 29. This adjustment compensates for the lost high-dimensional information, such as the distance between the clusters. The center *CC*_*r*_ of the Cluster *r* is adjusted to


$$\begin{array}{l} {CC}_{r}^{\text{adj}}=\alpha_{n}{CC}_{r}, \end{array}$$ (31)

$$\begin{array}{l} \rho_{n}=\frac{\Lambda_{n}}{\Lambda_{L}}, \end{array}$$ (32)

$$\begin{array}{l} \alpha_{n}=\frac{1}{\rho_{n}}, \end{array}$$ (33)

where ${CC}_{r}^{\text{adj}}$, ρ_*n*_, and α_*n*_ are the adjusted *CC*_*r*_, the cumulative contribution ratio, and correction coefficient, respectively. The information loss rate is represented by 1−ρ_*n*_. For example, when ρ_*n*_ = 0.8, 20% of the high-dimensional information is lost. Therefore, in **D**_w_(*t*), the distances between the clusters are larger. To compensate for the lost information, *CC*_*r*_ is multiplied by α_*n*_. After adjusting the centers of all clusters, the box-counting, lacunarity, and mass-radius methods are applied to ${\text{G}}_{\text{w}}^{\text{adj}}\left(t\right)$ to estimate the fractal dimension.

Finally, based on the results obtained from numerical simulation experiments, we reproduce the fractal structure formed in the synaptic weight space using an IFS. In numerical experiments, the input patterns increase exponentially with the number of spatial vectors *K* and their length *T*; thus, it is difficult to simulate and observe the detailed structure of the distance between the synaptic weights. Therefore, an IFS is used to reproduce the time evolution of distances between the synaptic weights obtained from the numerical simulation experiments, enabling observation of fine-scale structures and confirming that the STLR forms a fractal structure. We describe an IFS that operates on **g**(*t*), the two-dimensional representation of the synaptic weights **W**(*t*) obtained through the MDS method as


$$\begin{array}{lll} \text{W}\left(t\right)\in {\mathbb{R}}^{N\times M} & \overset{\text{MDS}}{\to } & \text{g}\left(t\right)\in {\mathbb{R}}^{2}, \end{array}$$ (34)

$$\begin{array}{l} \text{g}\left(t+1\right)=\overset{K}{\underset{k}{⋃}}F_{k}\left(\text{g}\left(t\right)\right), \end{array}$$ (35)

$$\begin{array}{l} F_{k}\left(\text{g}\left(t\right)\right)=\text{g}\left(t\right)+\gamma_{k}\left(t\right)\left[0\;1\right] \end{array}$$

$$\begin{array}{l} \;\left[\begin{array}{cc} \cos\left(\phi_{k}\left(t\right)\right) & \sin\left(\phi_{k}\left(t\right)\right)\\ -\sin\left(\phi_{k}\left(t\right)\right) & \cos\left(\phi_{k}\left(t\right)\right) \end{array}\right], \end{array}$$ (36)

where *F*_*k*_ is a mapping function when **z**^*k*^ is input. γ_*k*_(*t*) and ϕ_*k*_(*t*) is an expansion coefficient and a phase angle based on **g**(*t*) at *k* = 1, ϕ_1_(*t*) = 0, respectively. Furthermore, the vector [0 1] is multiplied to keep the mapping symmetric with respect to the y-axis. The IFS is constructed such that the geometric relationships in the low-dimensional space **g**(*t*) reflect the distance in the high-dimensional synaptic weights space **W**(*t*). For instance, if mapping functions *F*_1_ and *F*_2_ transform **g**(*t*), the resulting Euclidean distance between **g**^1^(*t*+1) and **g**^2^(*t*+1) corresponds to the Euclidean distance between the updated weights **W**^1^(*t*+1) and **W**^2^(*t*+1).

## 3 Results

In the following numerical simulations, the parameter values are set as *N* = 120, *M* = 120, η = 2, θ_LTP_ = 4, θ_LTD_ = −2, and τ_Q_ = 2.23. These parameter values are the best learning performance in the previous studies (Tsuji et al., 2023a). Furthermore, these parameter values satisfy the necessary conditions for the network's synaptic weights to be updated (for details, see Supplementary Sections 1, 2, and 9).

### 3.1 Two-dimensional distance maps and cosine similarity analysis

The two-dimensional distance maps created using **D**_w_(*T*+1) and **D**_y_(*T*+1) are shown in Figures 2a and 2b, respectively. In Figures 2a and 2b, the vertical and horizontal axes represent the labels of the input spatiotemporal patterns.

In Figure 2a, the color bar represents *ED*(**W**^*l*^(*T*+1), **W**^*l*^′(*T*+1)). The higher values indicate the higher dissimilarity. When the network learns the same spatiotemporal pattern (i.e., *l* = *l*′), *ED*(**W**^*l*^(*t*), **W**^*l*^′(*t*)) = 0. By contrast, in Figure 2b, the color bar represents *HD*(**Y**^*l*^(*T*+1), **Y**^*l*^′(*T*+1)).

In Figure 2a, a yellow area appears at *ED*(**W**^*l*^(*T*+1), **W**^*l*^′(*T*+1))>400 when the network learns the different first spatial vectors in the input spatiotemporal patterns (i.e., when $k_{1}^{l}\neq k_{1}^{l^{\prime }}$). In contrast, three green rectangles appear at *ED*(**W**^*l*^(*T*+1), **W**^*l*^′(*T*+1))≈350 when the network learns the same first spatial vectors in the input spatiotemporal patterns (i.e., when $k_{1}^{l}=k_{1}^{l^{\prime }}$). Moreover, when the spatial vectors up to the second are the same (i.e., when $k_{1}^{l}=k_{1}^{l^{\prime }}$ and $k_{2}^{l}=k_{2}^{l^{\prime }}$), 3^2^ = 9 light-blue rectangles appear at *ED*(**W**^*l*^(*T*+1), **W**^*l*^′(*T*+1))≈200. Consequently, a fractal-like structure is confirmed in the distance space of the synaptic weights, which reflects the temporal order of the spatial vectors in the input spatiotemporal patterns.

Figure 2b further shows an order-nested structure similar to that observed in Figure 2a. This finding suggests that the output space structure represents that of the synaptic weight space. In other words, the outputs of the network can be used to evaluate the synaptic weights.

In Figure 2, the cosine similarity in Equation 23 is used to quantitatively analyze the similarity between the structures of the synaptic weights and outputs in the distance space, resulting in *CS*(*T*+1) = 0.959. The average and variance of the cosine similarity are 0.959 and 3.73 × 10^−5^, respectively, when the initial values of the synaptic weights are changed 1,000 times (Supplementary Figure S1), suggesting that the synaptic weights and outputs have similar structures in the distance space. Furthermore, even when the network parameter values are changed, the similarity between the synaptic weights and outputs in the distance space is high (for details, see Supplementary Section 3).

### 3.2 MDS analysis of fractal structures

**Figure 3:**
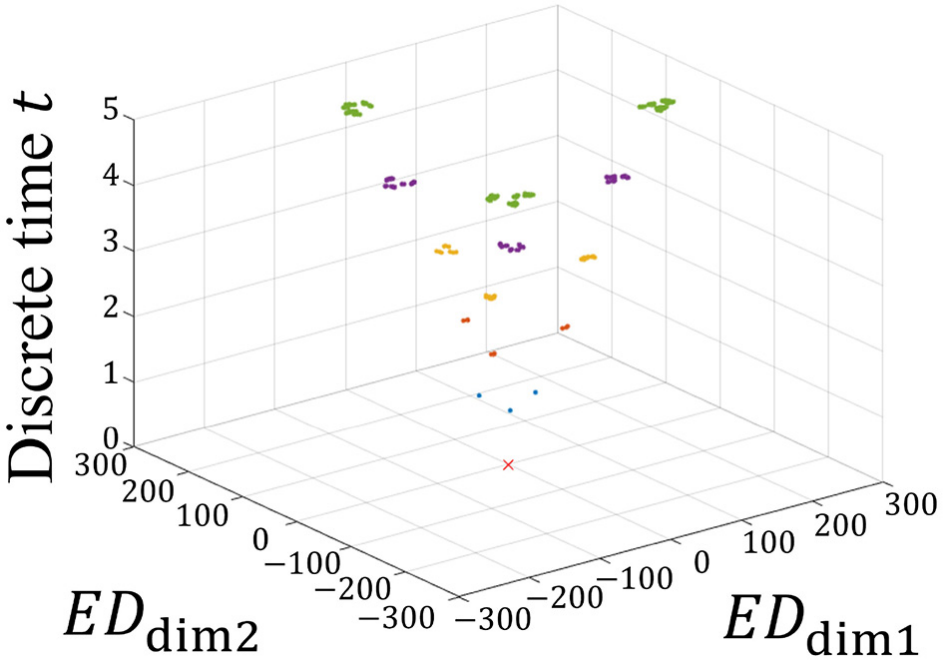
Time evolution of G_w_(*t*). *ED*_dim1_ and *ED*_dim2_ represent the distances in the dimension-compressed distance space. The vertical axis represents the discrete time *t*. The red × represents the initial state of G_w_(0) = 0 because all W^*l*^(0) are the same initial value. At times *t* = 1, 2, 3, 4, and 5, G_w_(*t*) are represented by blue, orange, yellow, purple, and green, respectively. As the network learns the spatiotemporal patterns, these states branch out sequentially at each time step, forming a hierarchical structure. This branching process visually demonstrates how the STLR progressively separates patterns, leading to the formation of a self-similar structure.

The MDS method (Torgerson, 1952) is applied to visualize **D**_w_(*t*) in a low dimension. A reasonable compression dimension *n* = 2 is obtained when Λ_*n*_≈0.5Λ_*L*_ (Supplementary Figure S5). Figure 3 shows the time evolution of **G**_w_(*t*) obtained by compressing **D**_w_(*t*) into two dimensions.

In Figure 3, the two horizontal and vertical axes represent the distances of **G**_w_(*t*) and the discrete time *t*, respectively. In the horizontal axes, *ED*_dim1_ and *ED*_dim2_ represent the first and second distances in the dimension-compressed distance space, respectively. At time *t* = 0, red × indicates the initial positions of the synaptic weights in the dimension-compressed distance space. For the 243 spatiotemporal patterns, the initial values of the synaptic weights are set to the same values, and all positions of **G**_w_(0) are (*ED*_dim1_, *ED*_dim2_) = (0, 0). Subsequently, at time *t* = 1, the red × branches to the three blue points in the figure because three different spatial vectors exist in the spatiotemporal pattern. Furthermore, branching occurs at *t*≥2. Assuming that this branching occurs repeatedly, this suggests that a self-similar structure is formed in the distance space. In other words, we propose that a fractal structure is formed in the synaptic weight space.

**Figure 4:**
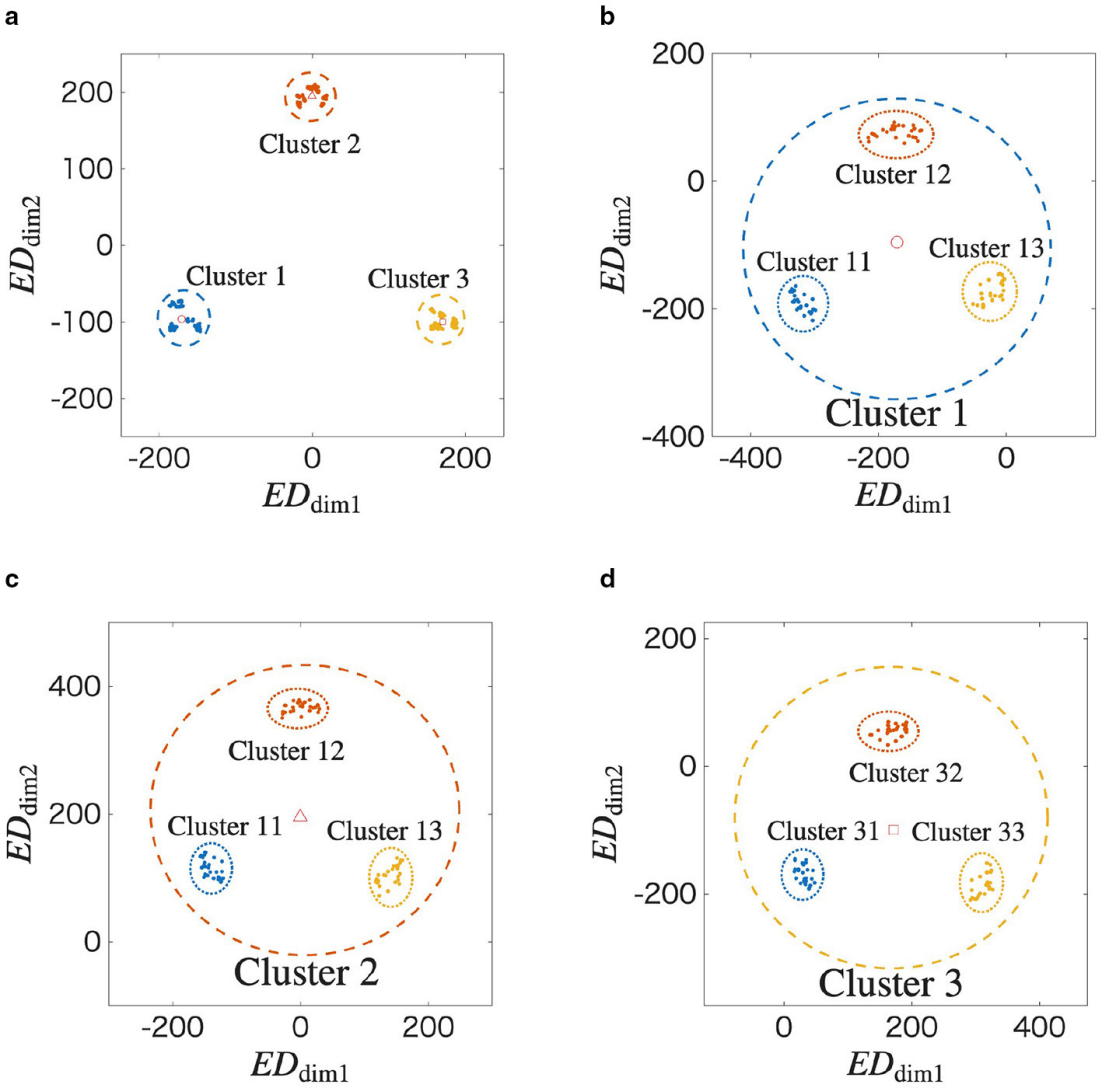
Recursive analysis of the final learned structure at *t* = 5, revealing its self-similar and scale-invariant properties. **(a)** A two-dimensional view of the final state from Figure 3. The data points are grouped into three clusters based on the first spatial vector in their respective input sequences (Cluster 1, 2, and 3). The center of each cluster is marked. **(b–d)** are applied the MDS to the Cluster 1, 2, and 3 in Figure 4a, respectively. Figures 4b–d reveal that the internal structure of a cluster is itself composed of three smaller sub-clusters, formed in a similar triangle. These sub-clusters are formed based on the second spatial vector in the input sequences. The repetition of the same geometric pattern at a smaller scale is a clear demonstration of the fractal properties of self-similarity and scale-invariance.

We further analyze the structure shown in Figure 3 in detail using the MDS method, repeatedly applying it according to the temporal order of the input spatiotemporal patterns. Figure 4a shows **G**_w_(*t*) at *t* = 5 from Figure 3.

In the figure, **g**^*l*^(*t*) is labeled as *r* according to $k_{1}^{l}$. In other words, Clusters *r* = 1, 2, and 3 when the network learns **X** = (**z**^1^, ⋯ ), (**z**^2^, ⋯ ), and (**z**^3^, ⋯ ), respectively. In Figure 4a, ○, ◇, and □ indicate the centers of each cluster, where (*ED*_dim1_, *ED*_dim2_) = (−171, −100), (0, 196), and (171, −96), respectively. We apply the MDS method to Clusters 1, 2, and 3. The results are shown in Figures 4b–d. In Figure 4b, ○ represents the center of Cluster 1 in Figure 4a. Similarly, in Figures 4c and 4d, ◇ and □ represent the centers of Clusters 2 and 3 in Figure 4a, respectively. In Figures 4b–d, **g**^*l*^(*t*) is labeled according to $k_{2}^{l}$. For example, Cluster *r* = 13 in Figure 4b represents the case when the network learns **X** = (**z**^1^, **z**^3^, ⋯ ).

Based on the above results, the spatiotemporal pattern of the input is memorized as a self-similar structure in the synaptic weight space. Additionally, scale-invariance can be confirmed because the triangular structure shown in Figure 4a also appears in Figures 4b–d. As a result, we qualitatively confirm that the STLR forms a fractal structure on the synaptic weight space.

Additionally, we analyzed the structure in the distance space between synaptic weights using the Isomap (Tenenbaum et al., 2000) and UMAP (McInnes et al., 2020) methods. The results are shown in Supplementary Figures S6 and S7, respectively. The Isomap and UMAP methods map high-dimensional information to a low-dimensional space while preserving as much of the original information as possible. These methods utilize *k*_NN_-nearest neighbors, and by adjusting the value of *k*_NN_, it is possible to investigate structures from local to global. However, even using these methods, it was not possible to reveal in detail the local and global structures shown in Figure 6. This is because of the significant difference in distance scales between local and global clusters.

**Figure 5:**
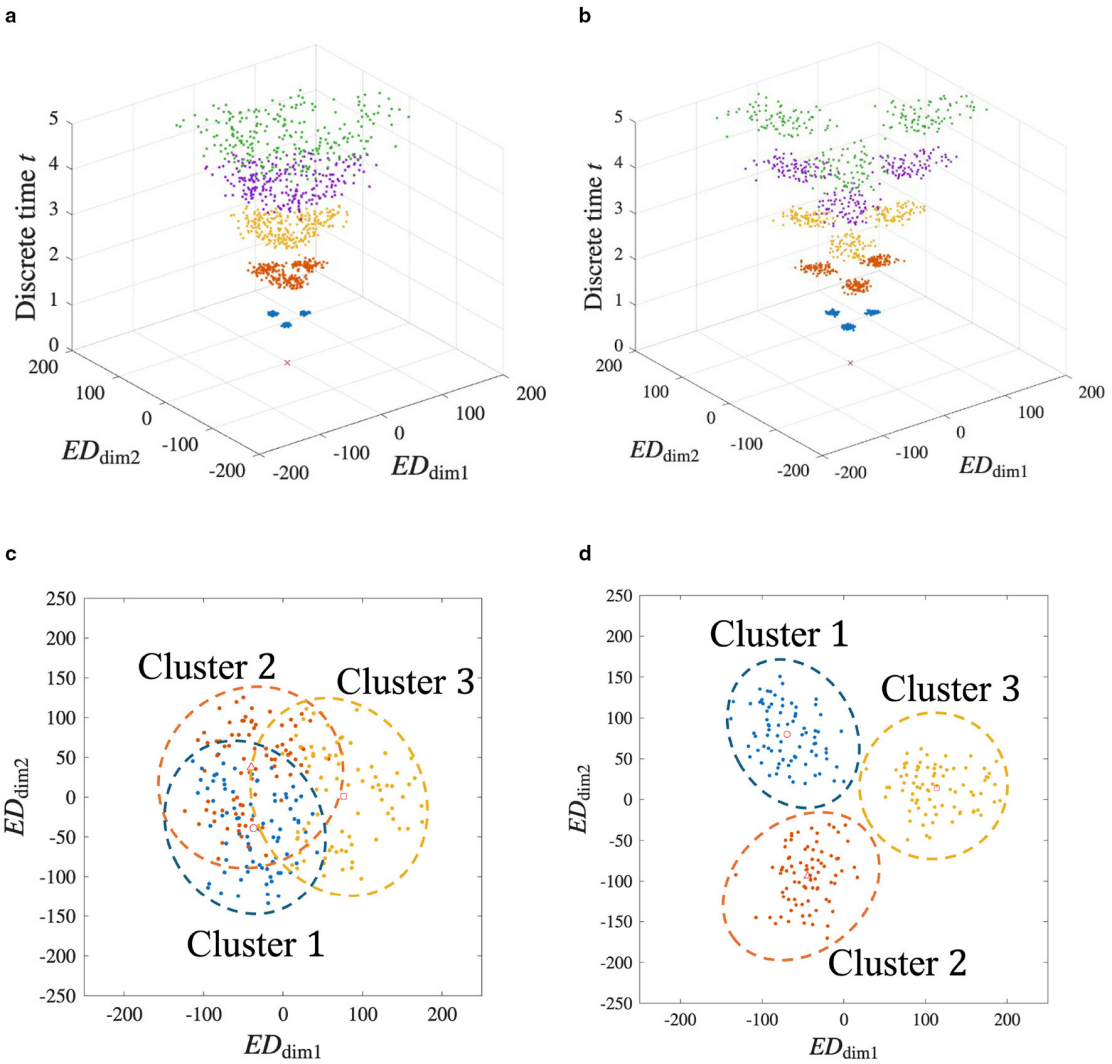
MDS analysis of the synaptic weight space under noisy input conditions. This figure demonstrates the network's robustness to noise and the role of the LTP threshold. White noise with an amplitude of σ_*p*_ is added to the inputs during learning. **(a)** and **(c)** Results for θ_LTP_ = 4. Figure 5a and 5c show the time evolution of the structure and the final state at *t* = 5, respectively. Figure 5c indicates the significant overlap between clusters. **(b)** and **(d)** Results for θ_LTP_ = 2. The time evolution **(b)** and final state **(d)** show that lowering the threshold results in better-defined, more clearly separated clusters. This suggests that a lower θ_LTP_ enhances the network's ability to handle noisy inputs, allowing it to improve the overall separation accuracy.

Figure 5 shows the time evolution and the structure of **G**_w_(*t*) when a white noise signal of amplitude σ_*p*_ is applied to the input (for results when the amplitude is changed to values other than σ_*p*_, see Supplementary Section 7). Figures 5a, c show the time evolution and the resulting structure at *t* = 5, respectively, when θ_LTP_ = 4. Similarly, Figures 5b, d show the results when θ_LTP_ = 2. When noise is high, decreasing θ_LTP_ enhances the network's ability to handle noisy inputs, improving the pattern separation accuracy of the clusters, which mitigates the cluster overlap seen in Figure 5c, as shown in Figure 5d. A decrease in θ_LTP_ caused an increase in the number of updated synapses. As a result, a noisy spatial vector is complemented. Therefore, it can be suggested that the ability to separate spatiotemporal patterns with noise is improved. The relationship between θ_LTP_ and the number of updated synapses is detailed in Supplementary Section 2.

### 3.3 Estimation of fractal dimension

To estimate the fractal dimension in Figure 4, **G**_w_(*t*) is adjusted using ρ_*n*_. The centers of each Cluster *r* are adjusted using Equation 33. For example, in Figure 4a, ${CC}_{r}^{\text{adj}}=2.2{CC}_{r}$, where *n* = 2; *r* = 1, 2, 3; ρ_*n*_ = 0.46; and α_*n*_ = 2.2. That is, ${CC}_{1}^{\text{adj}}$, ${CC}_{2}^{\text{adj}}$, and ${CC}_{3}^{\text{adj}}$ are (−374, −220), (0, 430), and (374, −210), respectively. **G**_w_(*t*) is adjusted to ${\text{G}}_{\text{w}}^{\text{adj}}\left(t\right)$ using α_*n*_ as shown in Table 2.

**Table 2:** Correction coefficients used for adjusting cluster coordinates in the MDS visualization.

**Cluster label *r***	**α_*n*_**
1, 2, 3	2.2
11, 12, ⋯ , 33	1.6
111, 112, ⋯ , 333	1.4
1111, 1112, ⋯ , 3333	1.3
11111, 11112, ⋯ , 33333	1

**Figure 6:**
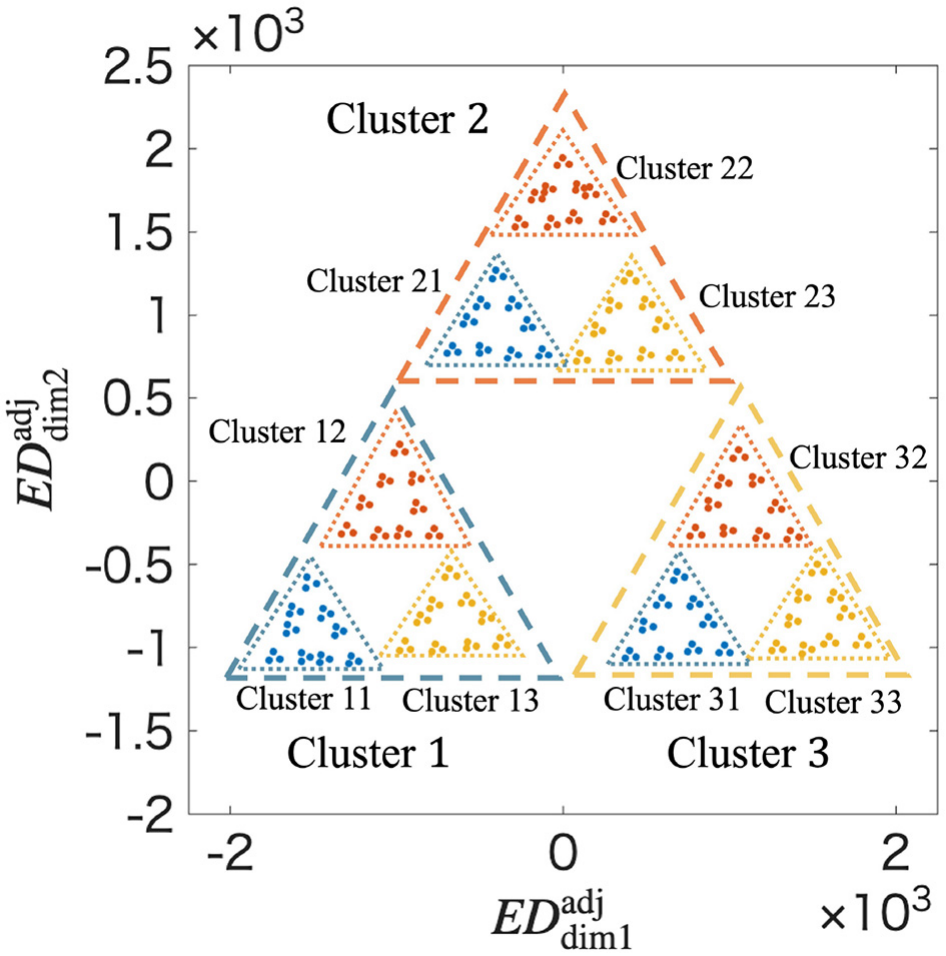
Fractal structure in ${\text{G}}_{\text{w}}^{\text{adj}}\left(t\right)$ at *t* = 5 using ρ_*n*_ obtained from the MDS. Clusters are classified according to the temporal order of spatial vectors up to the second in the input spatiotemporal patterns. ${ED}_{\text{dim}1}^{\text{adj}}$ and ${ED}_{\text{dim}2}^{\text{adj}}$ represent the distances in the adjusted distance space. The adjustment geometrically arranges the clusters according to their true distances in the high-dimensional space. The resulting visualization reveals a clear and detailed fractal structure, closely resembling a Sierpinski gasket. This adjusted representation allows for a more accurate estimation of the fractal dimension and confirms the hierarchical self-similarity of the memory structure.

The correction coefficients applied to adjust the cluster center coordinates for the visualization presented in Figure 6. These coefficients are calculated as the inverse of the cumulative contribution ratio of the eigenvalues obtained from the MDS analysis at each cluster. The “Cluster label *r*” denotes the hierarchical depth, with “1, 2, 3” representing the top-level clusters and “11, 12, ..., 33” representing the sub-clusters at the next level.

Figure 6 shows ${\text{G}}_{\text{w}}^{\text{adj}}\left(t\right)$ at *t* = 5. In Figure 6, a fractal structure similar to a Sierpinski gasket is confirmed, which could not be observed in Figure 4. The fractal dimension is determined to be 1.415 by applying the box-counting method to ${\text{G}}_{\text{w}}^{\text{adj}}\left(t\right)$ shown in Figure 6 (Supplementary Figure S14). Similarly, the average of the coefficient of variation and the averag of local fractal dimension are 0.6048 and 1.321 by applying the lacnarity and mass-radius methods, respectively (Supplementary Figures S15 and S17). These results quantitatively confirm that a fractal structure is formed in the synaptic weight space through the STLR.

### 3.4 Reproduction of fractal structure using IFS

**Figure 7:**
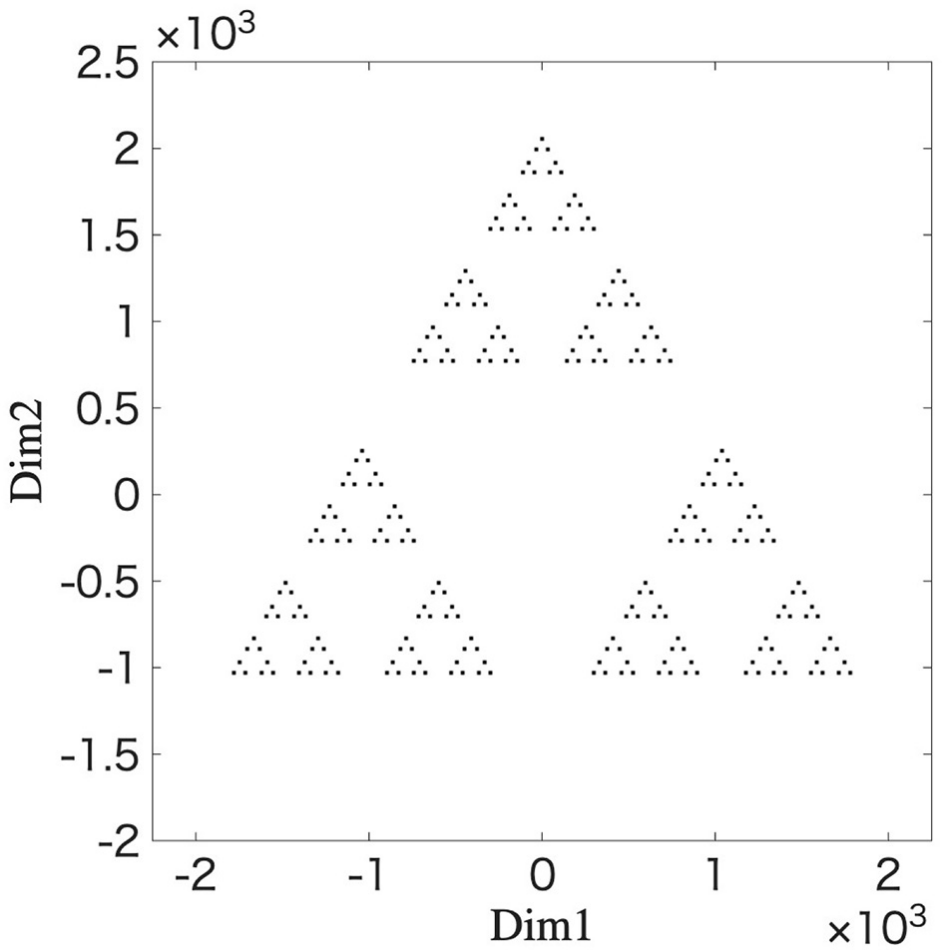
Fractal structure generated by an iterated function system. The simulation results are plotted **g**(*t*) at *t* = 5 after 2,000 iterations of the mapping 0 ≤ *t* ≤ 5 using Equation 35. The parameters for the IFS (expansion coefficient and phase angles) are estimated from the branching behavior observed in the network simulation (Figure 3).

We utilize the IFS to reproduce the fractal structure in the distance space between the synaptic weights. The initial values and parameter values for the IFS in Equations 35 are as follows: **g**(0) = [0 0], γ_*k*_(0) = 1200, γ_*k*_(*t*+1) = 0.424γ_*k*_(*t*), ϕ_1_(*t*) = 0, ϕ_2_(*t*) = 2π/3, and ϕ_3_(*t*) = −2π/3, respectively. These parameter values are obtained from the branching behavior observed in the network simulation as shown in Figure 3 (for details, see Supplementary Section 10). Functions *F*_1_, *F*_2_, and *F*_3_ are selected with a 1/3 probability using uniform random numbers. Equation 35 is repeated for 0 ≤ *t* ≤ 5. Figure 7 shows the results of **g**(*t*) at *t* = 5 after repeating the above procedure 2,000 iterations. The resulting structure's striking resemblance to the fractal structure shown in Figure 6 provides strong evidence that the learning process of the STLR can form a fractal structure. This provides a mathematical basis for the fractal coding being performed through the STLR.

In conclusion, a single-layer feedforward neural network with the STLR synapses memorizes the input spatiotemporal patterns as the fractal structure in the synaptic weight space.

## 4 Discussion

### 4.1 Fractal structure in the STLR

In Section 3.3, we analyzed the fractal structure in the distance space between the synaptic weights using the box-counting, lacunarity, and mass-radius methods (Supplementary Figures S14–S17) by the MDS method, as shown in Figure 4. The coefficient of variation and the distribution of the local fractal dimension shown in Supplementary Figures S15 and S17, respectively, suggest that the fractal structure is inhomogeneous.

However, the rigorous multifractal formalism established by Halsey et al. (1986) analyzes the scaling properties of a measure defined on that set, rather than the geometric distribution of the set itself. Our analysis focused only on geometric information, and a strict multifractal analysis has not yet been performed. This points to a critical issue for future research.

By deriving the generalized dimensions and the singularity spectrum within the rigorous multifractal formalism, it is possible to characterize the STLR's learning properties. The value of the generalized dimension allows for the analysis of different parts of a measure. A crucial future task is to quantitatively discuss how the memory structure formed by the STLR balances these conflicting functions of pattern completion and separation, as shown in Figure 5, by analyzing the shape of its multifractal spectrum.

### 4.2 Fractal coding through the STLR

Fractal coding encodes information into hierarchical self-similar structures as shown in Figure 4. The spatiotemporal patterns are encoded into a static synaptic weight space through the STLR and read out in the output space by inputting a specific signal in a single shot. Therefore, fractal coding through the STLR enables highly efficient compression and processing of information. On the other hand, for the Cantor coding proposed in the literature (Tsuda, 2001; Yamaguti et al., 2011), the spatiotemporal patterns are dynamically encoded into the state space of a network and read out as a time-series by inputting a specific signal. However, fractal coding using the STLR also dynamically encodes and reads out information by introducing recurrent connections into the network (Tsukada and Tsukada, 2021b).

The pattern separation ability of the STLR significantly contributes to the fractal coding. The STLR can learn slight differences in the similar spatiotemporal patterns. Therefore, clusters and hierarchical structures were formed based on the spatial and temporal differences in the spatiotemporal patterns using the STLR, respectively. Consequently, the spatiotemporal patterns were memorized as a fractal structure.

For the pattern separation ability of the STLR, we suggest that the learning rate η, the firing rate of the input spatial vectors (the ratio of 0s to 1s), the time constant τ_Q_, LTP threshold θ_LTP_, and LTD threshold θ_LTD_ are important parameters from preliminary experiments. In the present study, these parameter values were obtained from (Tsuji et al., 2023a), where the order-nested nested structure in the output is clearly observable through an exhaustive search. However, these parameter values should be determined in accordance with physiological findings (Tsukada et al., 1996) to reproduce physiological experiments. In particular, θ_LTP_ and θ_LTD_ are important for learning the spatial structure of the spatiotemporal patterns.

From a physiological perspective, the balance between these thresholds is hypothesized to be related to the concentration of neuromodulators such as acetylcholine. The relationship between the thresholds controls the trade-off between pattern completion and pattern separation. Under the condition θ_LTD_≪θ_LTP_≈μ_*q*_, the learning of common features within an input sequence becomes dominant, strengthening the characteristics of pattern completion and contributing to the convergence to an attractor through fractal structuring. Conversely, under the condition θ_LTD_≈μ_*q*_≪θ_LTP_, the learning of common elements is canceled out, and the system becomes dominated by the learning of orthogonal elements, thereby strengthening pattern separation. This suggests that the hippocampus forms memories based on an appropriate balance between these two abilities, a balance that may be modulated by acetylcholine. Furthermore, this suggestion aligns with the results of the noise-added experiments in Section 3.2. This perspective is important as it has implications for physiological experimental research.

Based on the conditions derived in Supplementary Section 2, if θ_LTP_ is too large and θ_LTD_ is too small, the synaptic weight values are hardly updated (i.e., the network does not learn the spatiotemporal patterns) and clusters are not formed. Additionally, τ_Q_ significantly affects the learning of the temporal structure of the spatiotemporal patterns. If τ_Q_ is too small, learning the temporal structure is difficult owing to the influence of the initial values of the synaptic weights. Therefore, it is necessary to determine these parameter values based on the results of physiological experiments. Numerical simulations using these parameter values enabled analyses that are difficult to perform in physiological experiments. Furthermore, a theoretical approach is required to elucidate the learning mechanisms of the STLR in detail.

### 4.3 Interrelationship between the structures in the synaptic weight and output spaces

The relationship between the fractal structure in the synaptic weight space and the order-nested structure in the output space is crucial, as the output space provides a physiological link to an organism's behavior. We propose that the sensory memory structure of a recognition system is encoded as a fractal structure in the synaptic weight space, and this structure can be expressed in the output space as needed to inform behavior.

In a previous study, we identified the order-nested structures formed in the distance space of the network's outputs with the STLR synapses (Tsuji et al., 2023a). Figure 2 confirms that the distance map created from synaptic weight values has a fractal structure. To clarify the interrelationship between these structures, we performed numerical simulations by changing the initial values of the synaptic weights 1,000 times when *T* = 3 (see Supplementary Figure S2). Additionally, even when the network parameters are changed, the similarity between the synaptic weights and outputs in the distance space is high (for details, see Supplementary Section 3).

Histograms and distance maps of the synaptic weights, internal states, and outputs were created, respectively, for each initial value (Supplementary Figure S4). Consequently, a fractal structure was observed in the synaptic weight space for all the initial conditions. By contrast, the order-nested structures were not always observed in the internal state and output spaces. In other words, the fractal structure in the weight space does not always appear in the output space. There are two possible reasons for this. The first is the dimensional compression from the input to the internal state. The inputs are multiplied by the weights and spatially summed and averaged in the internal state. Consequently, the information embedded in the synaptic weight space is compressed from ℝ^*M*×*N*^ to ℝ^*N*^. The second is the binarization of the internal state at the output through thresholding. This further compresses information in the internal state (i.e., ℝ^*N*^ → {0, 1}^*N*^). If the firing threshold is not properly adjusted, synaptic weight information is further reduced. Conversely, our simulations showed that when an order-nested structure appears in the output space, a fractal structure is always found in the synaptic weight space. This is because synaptic weights always form a fractal structure (Supplementary Section 9) when the parameter values satisfy the conditions derived in Supplementary Section 2. Consequently, we concluded that the order-nested structure in the output space guarantees a fractal structure in the synaptic weight space. However, the fundamental reason why the order-nested structure does not emerge in the output space remains unexplained. We suggest that the initial values of the synaptic weights have a significant influence on this issue. As mentioned in Supplementary Section 10, this network exhibits chaotic behavior, and this is due to its characteristic sensitivity to initial conditions. Therefore, future research will analyze the chaotic behavior of synaptic weights and the conditions under which the order-nested structure appears in the output.

### 4.4 Hardware implementation

Recently, neural-network-based artificial intelligence (AI) has been applied to a variety of fields (Maslej et al., 2024). However, several problems have arisen in this regard. For example, the large-language models consume a large amount of power and data for learning, causing serious social problems (Team, 2025). This indicates that current AI models are not suitable for applications where energy, area, and training data are restricted, such as edge-devices.

In contrast, a neural network based on the STLR can learn contexts embedded in the spatiotemporal sequences with a small number of examples. Furthermore, the STLR uses only local information around each synapse; thus, a small area is sufficient for hardware implementation. Additionally, because the STLR is inherently asynchronous (Orima et al., 2023), it is suitable for spiking neural networks (Tsuji et al., 2024), which drastically reduces power consumption. From a hardware perspective, fractal coding achieves hierarchical spatiotemporal information compression, resulting in highly efficient memory usage. Therefore, the STLR is a good candidate for the integrated circuit implementation of small-sized, efficient, and high-performance spiking neural networks with one-shot learning capability (Orima et al., 2023). Furthermore, we investigated circuit precision requirements for hardware implementation and found that the original STLR model can be reproduced even with approximately 8-bit circuit implementation (Tsuji et al., 2023b). Based on this result, we are developing a dedicated hardware emulator for designing analog integrated circuits (Tsuji et al., 2024). Using this emulator, we are investigating parameter values for the implementation of analog integrated circuits and verifying the robustness of the system, considering device variations.

However, it is hard to measure the synaptic weight values from an integrated circuit because the state of each synaptic circuit/device is usually inaccessible, and if possible, the number of synapses is unpractically large. Therefore, evaluating the learning characteristics based on the synaptic weight space is difficult. That is, the fractal structure in the synaptic weight space resulting from the STLR, which was evaluated using simulations, could not be experimentally observed from the integrated circuit measurements. However, as discussed in Section 4.3, an order-nested structure in the output space provides a good measure of the fractal structure in the synaptic weight space. Consequently, the outputs of the network, which are easily measurable, can be used to evaluate fractal coding through the STLR in integrated circuit systems.

## Data Availability

The raw data supporting the conclusions of this article will be made available by the authors, without undue reservation.

## References

[B1] Bieberich E. (2002). Recurrent fractal neural networks: a strategy for the exchange of local and global information processing in the brain. BioSystems 66, 145–164. doi: 10.1016/S0303-2647(02)00040-0 12413746

[B2] Fukushima Y. Tsukada M. Tsuda I. Yamaguti Y. Kuroda S. (2007). Spatial clustering property and its self-similarity in membrane potentials of hippocampal CA1 pyramidal neurons for a spatio-temporal input sequence. Cogn. Neurodyn. 1, 305–316. doi: 10.1007/s11571-007-9026-9 19003501 PMC2289047

[B3] Guardamagna M. Stella F. Battaglia F. P. (2023). Heterogeneity of network and coding states in mouse CA1 place cells. Cell Rep. 42:112022. doi: 10.1016/j.celrep.2023.112022 36709427

[B4] Halsey T. C. Jensen M. H. Kadanoff L. P. Procaccia I. Shraiman B. I. (1986). Fractal measures and their singularities: the characterization of strange sets. Phys. Rev. A 33, 1141–1151. doi: 10.1103/PhysRevA.33.1141 9896729

[B5] Hebb D. O. (1949). The Organization of Behavior. New York: Wiley.

[B6] Husain A. Nanda M. N. Chowdary M. S. Sajid M. (2022). Fractals: an eclectic survey, part-I. Fractal Fract. 6:89. doi: 10.3390/fractalfract6020089

[B7] Kovcs K. A. (2020). Episodic memories: how do the hippocampus and the entorhinal ring attractors cooperate to create them? Front. Syst. Neurosci. 14:559186. doi: 10.3389/fnsys.2020.559186 33013334 PMC7511719

[B8] Kuroda S. Fukushima Y. Yamaguti Y. Tsukada M. Tsuda I. (2009). Iterated function systems in the hippocampal CA1. Cogn. Neurodyn. 3, 205–222. doi: 10.1007/s11571-009-9086-0 19554477 PMC2727166

[B9] Landini G. Rippin J. W. (1993). Notes on the implementation of the mass – radius method of fractal dimension estimation. Bioinformatics 9, 547–550. doi: 10.1093/bioinformatics/9.5.547 8293328

[B10] Mandelbrot B. B. (1982). The Fractal Geometry of Nature. San Francisco, CA: Freeman.

[B11] Mandelbrot B. B. (1994). A Fractal's Lacunarity, and How It Can Be Tuned and Measured. Basel: Birkhäuser Basel.

[B12] Maslej N. Fattorini L. Perrault R. Parli V. Reuel A. Brynjolfsson E. . (2024). Artificial Intelligence Index Report 2024. Available online at: https://arxiv.org/abs/2405.19522

[B13] McCloskey M. Cohen N. J. (1989). Catastrophic interference in connectionist networks: the sequential learning problem. Psychol. Learn. Motiv. 24, 109–165. doi: 10.1016/S0079-7421(08)60536-8

[B14] McInnes L. Healy J. Melville J. (2020). UMAP: Uniform Manifold Approximation – *and Projection for Dimension Reduction*. Available online at: https://arxiv.org/abs/1802.03426

[B15] Melchior J. Altamimi A. Bayati M. Cheng S. Wiskott L. (2024). Correction: a neural network model for online one-shot storage of pattern sequences. PLoS ONE 19:e0313130. doi: 10.1371/journal.pone.0313130 38900733 PMC11189254

[B16] Orima T. Tsuji T. Horio Y. (2023). An extended spatiotemporal contextual learning and memory network model for hardware implementation. Procedia Comp. Sci. 222, 478–487. doi: 10.1016/j.procs.2023.08.186

[B17] Rolls E. T. (2010). A computational theory of episodic memory formation in the hippocampus. Behav. Brain Res. 215, 180–196. doi: 10.1016/j.bbr.2010.03.027 20307583

[B18] Sarkar N. Chaudhuri B. (1994). An efficient differential box-counting approach to compute fractal dimension of image. IEEE Trans. Syst. Man Cybern. 24, 115–120. doi: 10.1109/21.259692

[B19] Smith T. Lange G. Marks W. (1996). Fractal methods and results in cellular morphology – dimensions, lacunarity and multifractals. J. Neurosci. Methods 69, 123–136. doi: 10.1016/S0165-0270(96)00080-5 8946315

[B20] Team ICS (2025). AI and Resource Consumption: Examining the Environmental Impact. Available online at: https://www.computer.org/publications/tech-news/research/ai-and-the-environment

[B21] Tenenbaum J. B. de Silva V. Langford J. C. (2000). A global geometric framework for nonlinear dimensionality reduction. Science 290, 2319–2323. doi: 10.1126/science.290.5500.2319 11125149

[B22] Torgerson W. S. (1952). Multidimensional scaling: I. theory and method. Psychometrika 17, 401–419. doi: 10.1007/BF02288916 5217606

[B23] Tsuda I. (2001). Toward an interpretation of dynamic neural activity in terms of chaotic dynamical systems. Behav. Brain Sci. 24, 793–848. doi: 10.1017/S0140525X01000097 12239890

[B24] Tsuji T. Orima T. Horio Y. (2023a). “Detailed evaluation of spatiotemporal learning rule based on hamming distances among output vectors,” in The 2023 International Symposium on Nonlinear Theory and Its Applications (NOLTA) [The Institute of Electronics, Information and Communication Engineers (IEICE)], 415–418.

[B25] Tsuji T. Orima T. Horio Y. (2023b). “A study on circuit accuracy for hardware implementations of spatio-temporal learning rule,” in Proc. IEICE Gen. Conf . (*in Japanese)* [The Institute of Electronics, Information and Communication Engineers (IEICE)].

[B26] Tsuji T. Orima T. Horio Y. (2024). “An event-driven mixed analog/digital spiking neural network circuit model for hippocampal spatiotemporal context learning and memory,” in 2024 International Joint Conference on Neural Networks (IJCNN) [The Institute of Electronics, Information and Communication Engineers (IEICE)], 1–8.

[B27] Tsukada H. Tsukada M. (2021a). Comparison of pattern discrimination mechanisms of hebbian and spatiotemporal learning rules in self-organization. Front. Syst. Neurosci. 15:624353. doi: 10.3389/fnsys.2021.624353 33854419 PMC8039312

[B28] Tsukada H. Tsukada M. (2021b). “Context-dependent learning and memory based on spatio-temporal learning rule,” in Advances in Cognitive Neurodynamics, eds. A. Lintas, P. Enrico, X. Pan, R. Wang, and A. Villa (Singapore: Springer Singapore), 89–94.

[B29] Tsukada M. Aihara T. Saito H. Kato H. (1996). Hippocampal ltp depends on spatial and temporal correlation of inputs. Neural Netw. 9, 1357–1365. doi: 10.1016/S0893-6080(96)00047-0 12662539

[B30] Tsukada M. Pan X. (2005). The spatiotemporal learning rule and its efficiency in separating spatiotemporal patterns. Biol. Cybern. 92, 139–146. doi: 10.1007/s00422-004-0523-1 15696314

[B31] Werner G. (2010). Fractals in the nervous system: Conceptual implications for theoretical neuroscience. Front. Physiol. 1:15. doi: 10.3389/fphys.2010.00015 21423358 PMC3059969

[B32] Yamaguti Y. Kuroda S. Fukushima Y. Tsukada M. Tsuda I. (2011). A mathematical model for cantor coding in the hippocampus. Neural Netw. 24, 43–53. doi: 10.1016/j.neunet.2010.08.006 20850269

