# Supplementary material for: Fractal memory structure in the spatiotemporal learning rule
Source: Front Comput Neurosci. 2025 Dec 16;19:1641519. doi: 10.3389/fncom.2025.1641519 (PMC12748222; doi:10.3389/fncom.2025.1641519)
Supplement: Supplementary file 1 [file Data_Sheet_1.pdf]

Supplementary Material

1 MEAN AND VARIANCE OF SYNAPTIC WEIGHTS, INPUTS, INTERNAL STATES, AND INPUT COINCIDENCES

To analyze the network in detail, we calculate statistics for synaptic weights, inputs, internal states, and input concordances. In particular, the first input has a significant impact on network learning. If the first input does not update the synaptic weights, the probability that the network's synaptic weights will be updated is low, even when similar inputs are subsequently applied. Therefore, we calculate the network's statistics for the first input. Furthermore, the probability of updating the network's synaptic weights is explained in Supplementary Section 2.

At time $t = 1$, we calculate mean and variance in the synaptic weight $w_{ij}(t)$, input $x_j(t)$, internal state $p_i(t)$, and input coincidence $q_{ij}(t)$, respectively.

First, since $w_{ij}(1)$ and $x_j(1)$ are a continuous uniform random variable in the range $[-1, 1]$ and a discrete uniform random variable in the set $\{0, 1\}$, respectively, the mean and variance of $w_{ij}(1)$ and $x_j(1)$ are given by

$$\mu_w = E[w_{ij}(1)] = 0, \qquad \sigma_w^2 = V[w_{ij}(1)] = \frac{1}{3}, \tag{S1}$$

$$\mu_x = E[x_j(1)] = \frac{1}{2}, \qquad \sigma_x^2 = V[x_j(1)] = \frac{1}{4}, \tag{S2}$$

where μ_w and σ_w^2 represent the mean and variance of the synaptic weight, respectively; μ_x and σ_x^2 represent the mean and variance of the input, respectively; $E[z]$ and $V[z]$ are the mean and variance of the random variable z, respectively.

Next, the mean μ_p of $p_i(1)$ is

$$\mu_p = E[p_i(1)] = E\left[\sum_{j=1}^{M} w_{ij}(1)x_j(1)\right], \tag{S3}$$

where $w_{ij}(1)$ and $x_j(1)$ are independent random variables, respectively. Thus, μ_p is given by

$$\mu_p = E[w_{i1}(1)]E[x_1(1)] + \cdots + E[w_{iM}(1)]E[x_M(0)] = 0. \tag{S4}$$

Similarly, the variance σ_p^2 of $p_i(1)$ is

$$\begin{aligned}
\sigma_p^2 &= V[p_i(1)] \\
&= V[w_{i1}(1)x_1(1)] + \cdots + V[w_{iM}(1)x_M(1)] \\
&= V[w_{i1}(1)]V[x_1(1)] + V[w_{i1}(1)]\left(E[x_1(1)]\right)^2 + V[x_1(1)]\left(E[w_{i1}(1)]\right)^2 + \cdots \\
&\quad + V[w_{iM}(1)]V[x_M(1)] + V[w_{iM}(1)]\left(E[x_M(1)]\right)^2 + V[x_M(1)]\left(E[w_{iM}(1)]\right)^2 \\
&= \frac{M}{6}.
\end{aligned} \tag{S5}$$

Finally, when $q_{ij}(0) = 0$, the mean μ_q and variance σ_q^2 of $q_{ij}(1)$ are

$$\begin{aligned}
\mu_q &= E[q_{ij}(1)] = E[c_{ij}(1)] \\
&= E[w_{ij}(1)x_j(1)(p_i(1) - w_{ij}(1)x_j(1))] \\
&= E[w_{ij}(1)]E[x_j(1)]E[w_{i1}(1)]E[x_1(1)] + \cdots + E[w_{ij}(1)]E[x_j(1)]E[w_{iM}(1)]E[x_M(1))] \\
&= 0, \qquad \text{(S6)} \\
\sigma_q^2 &= V[q_{ij}(1)] = V[c_{ij}(1)] \\
&= V[w_{ij}(1)x_j(1)(p_i(1) - w_{ij}(1)x_j(1))] \\
&= V[w_{ij}(1)x_j(1)]V[w_{i1}(1)x_1(1)] + \cdots + V[w_{ij}(1)x_j(1)]V[w_{iM}(1)x_M(1)] \\
&= \frac{M-1}{36}. \qquad \text{(S7)}
\end{aligned}$$

2 PROBABILITY OF UPDATING SYNAPTIC WEIGHTS IN THE STLR

In the distance space of the synaptic weights, a necessary condition for forming a fractal structure is that the synaptic weights must be updated at $t = 1$. This is because if the synaptic weights are not updated at $t = 1$, they will not be updated for $t > 1$. Therefore, we derive the parameter values that ensure at least one synapse is updated at $t = 1$.

The probability of long-term potentiation (LTP) and long-term depression (LTD) at a synaptic weight $w_{ij}(t)$ are denoted as $P_{\mathrm{LTP}}(t, \theta_{\mathrm{LTP}})$ and $P_{\mathrm{LTD}}(t, \theta_{\mathrm{LTD}})$, respectively. At time t, $P_{\mathrm{LTP}}(t, \theta_{\mathrm{LTP}})$ and $P_{\mathrm{LTD}}(t, \theta_{\mathrm{LTD}})$ are derived from $q_{ij}(t)$ using LTP threshold θ_{LTP} and LTD threshold θ_{LTD}, respectively, as

$$P_{\mathrm{LTP}}(t, \theta_{\mathrm{LTP}}) = 1 - \int_{-\infty}^{\theta_{\mathrm{LTP}}} f^{\mathrm{prob}}(q_{ij}(t))\, \mathrm{d}q_{ij}(t), \qquad \text{(S8)}$$

$$P_{\mathrm{LTD}}(t, \theta_{\mathrm{LTD}}) = 1 - \int_{\theta_{\mathrm{LTD}}}^{\infty} f^{\mathrm{prob}}(q_{ij}(t))\, \mathrm{d}q_{ij}(t), \qquad \text{(S9)}$$

where $f^{\mathrm{prob}}(q_{ij}(t))$ is a probability density function (PDF) of $q_{ij}(t)$. However, $f^{\mathrm{prob}}(q_{ij}(t))$ is not easily derived. Thus, we derive $f^{\mathrm{prob}}(q_{ij}(t))$ when $t = 1$ by the following steps.

First, we approximate $q_{ij}(t)$ at $t = 1$ as

$$\begin{aligned}
q_{ij}(1) &= \sum_{t'=0}^{1} c_{ij}(t') \exp\left(-\frac{1-t'}{\tau_{\mathrm{Q}}}\right), \\
&= c_{ij}(1) + c_{ij}(0) \exp(-1/\tau_{\mathrm{Q}}), \qquad \text{(S10)}
\end{aligned}$$

where $q_{ij}(1) = c_{ij}(1)$ if $c_{ij}(0) = 0$. From Equation 4, $c_{ij}(1)$ is approximated as

$$c_{ij}(1) \approx w_{ij}(1)x_j(1)p_i(1), \qquad \text{(S11)}$$

where $p_i(1)$ is a normal distribution with mean $\mu_p = 0$ and variance $\sigma_p^2 = M/6$. When $x_j(1) = 1$, the PDF of $c_{ij}(1)$, denoted as $f_c^{\mathrm{prob}}(z)$, is derived using the following equation (Springer, 1979):

$$f_c^{\mathrm{prob}}(c_{ij}(1)) = \frac{1}{4}\int_{c_{ij}(1)}^{\infty} \frac{1}{z}\frac{1}{\sqrt{2\pi\sigma_p^2}} \exp\left\{-\frac{(z-\mu_p)^2}{2\sigma_p^2}\right\}\mathrm{d}z, \tag{S12}$$

where z is a random variable and $z > 0$. Therefore, $f^{\mathrm{prob}}(q_{ij}(t))$ of $q_{ij}(1)$ is defined as

$$f^{\mathrm{prob}}(q_{ij}(1)) = \begin{cases} f_c^{\mathrm{prob}}(|q_{ij}(1)|) & q_{ij}(1) \neq 0, \\ 1/2 & q_{ij}(1) = 0. \end{cases} \tag{S13}$$

Therefore, at time $t = 1$, from Equations S8 and S9, the probabilities of LTP and LTD are given by

$$P_{\mathrm{LTP}}(1, \theta_{\mathrm{LTP}}) = \begin{cases} 1 - \int_{-\infty}^{\theta_{\mathrm{LTP}}} f^{\mathrm{prob}}(q_{ij}(1))\mathrm{d}q_{ij}(t) & \theta_{\mathrm{LTP}} \neq 0, \\ 1/2 & \theta_{\mathrm{LTP}} = 0, \end{cases} \tag{S14}$$

$$P_{\mathrm{LTD}}(1, \theta_{\mathrm{LTD}}) = \begin{cases} 1 - \int_{\theta_{\mathrm{LTD}}}^{\infty} f^{\mathrm{prob}}(|q_{ij}(1)|)\mathrm{d}q_{ij}(t) & \theta_{\mathrm{LTD}} \neq 0, \\ 1/2 & \theta_{\mathrm{LTD}} = 0. \end{cases} \tag{S15}$$

Equations S14 and S15 are symmetric distributions at $q_{ij}(1) = \mu_q (= 0)$. Figure S1 shows the results of numerically integrating Equation S14. The horizontal axis in the figure represents $q_{ij}(1)$ and θ_{LTP}. The left and right vertical axes in the figure show $f^{\mathrm{prob}}(q_{ij}(1))$ of $q_{ij}(1)$ and $P_{\mathrm{LTP}}(1, \theta_{\mathrm{LTP}})$, respectively. In the figure, the histogram shows the probability of $q_{ij}(1)$ when the initial synaptic weights are changed 1000 times for $M = 120$ and $N = 120$. The result in Figure S1 shows that Equation S14 aligns well with the numerical simulation.

We determine θ_{LTP} and θ_{LTD} at which one or more synapses are updated in the M synapses of an ith neuron. When $MP_{\mathrm{LTP}}(1, \theta_{\mathrm{LTP}}) \geq 1$ or $MP_{\mathrm{LTD}}(1, \theta_{\mathrm{LTD}}) \geq 1$, at least one synaptic weight will be updated. Under this condition, the ranges of θ_{LTP} and θ_{LTD} are defined using the mean μ_q and variance σ_q^2 of $q_{ij}(1)$ as follows:

$$\mu_q - \beta\sigma_q \leq \theta_{\mathrm{LTD}} \leq \mu_q \leq \theta_{\mathrm{LTP}} \leq \mu_q + \beta\sigma_q, \tag{S16}$$

where β is a scaling coefficient, $\mu_q = 0$, and $\sigma_q^2 = (M-1)/36 \approx 3.3$ when $M = 120$. From Figure S1, $\theta_{\mathrm{LTP}} = 7.5$ when $MP_{\mathrm{LTP}}(1, \theta_{\mathrm{LTP}}) = 1$. Therefore, β is

$$\beta = \frac{\theta_{\mathrm{LTP}} - \mu_q}{\sigma_q} \approx 4. \tag{S17}$$

In conclusion, the range of thresholds satisfying $\mu_q - 4\sigma_q \leq \theta_{\mathrm{LTD}} \leq \mu_q \leq \theta_{\mathrm{LTP}} \leq \mu_q + 4\sigma_q$ is a necessary condition for forming a fractal structure.

3 COSINE SIMILARITY BETWEEN THE TWO-DIMENSIONAL DISTANCE MAPS

The distance matrices $\mathbf{D}_{\mathrm{w}}(t)$ and $\mathbf{D}_{\mathrm{y}}(t)$ are calculated using the synaptic weight matrix $\mathbf{W}^l(t)$ and output vector $\mathbf{Y}^l(t)$, respectively. To investigate the relationship between the synaptic weight space and output space using $\mathbf{D}_{\mathrm{w}}(t)$ and $\mathbf{D}_{\mathrm{y}}(t)$, the cosine similarity CS is calculated by Equation 24.

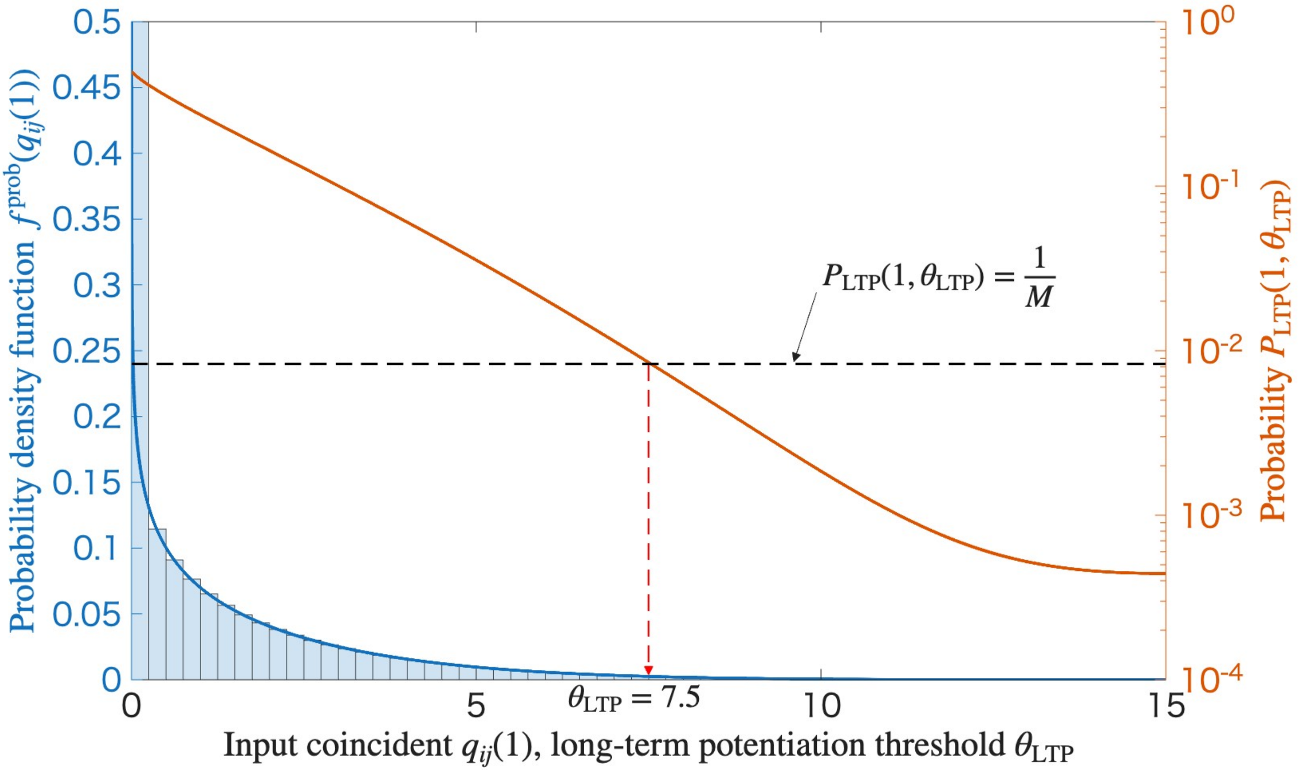

Figure S1. Probability density function of input coincidence and the probability of LTP. The horizontal axis represents $q_{ij}(1)$ and θ_{LTP}. The left vertical axis shows the PDF for the time history of coincidence. The solid blue line represents the result of numerically integrating Equation S12. The blue histogram shows the probability of $q_{ij}(1)$ when the initial synaptic weights are changed 1000 times for $M = 120$ and $N = 120$. The right vertical axis shows the probability of LTP. The black dashed line indicates the probability of updating one synapse when $M = 120$, which corresponds to $\theta_{\text{LTP}} \approx 7.5$.

Figure S2 shows the frequency of CS obtained when the initial values of the synaptic weights of the network are changed 1000 times. From the results in Figure S2, we confirm that the frequency distribution of CS approaches a normal distribution by the central limit theorem (Feller, 1968). The mean and variance of CS are 0.959 and 3.73×10^{-5}, respectively. Therefore, 99.7% of the CS values fall within the range of 0.94 to 0.977. These results show that the synaptic weight space and output space are highly similar in the distance space.

Additionally, Figure S3 shows the cosine similarity when the parameters θ_{LTP}, θ_{LTD}, τ_{Q}, and η are varied from 0 to $8\sigma_q$, from 0 to $-8\sigma_q$, with or without time history $\{0,\ 2.23\}$, and with small or large learning coefficients $\{0.5,\ 2\}$, respectively. The cosine similarity is the average value obtained by changing the initial synaptic weights 100 times. From the results in Figure S3, it is confirmed that when the parameter values of θ_{LTP} and θ_{LTD} satisfy the conditions determined in Supplementary Section 2, CS is high regardless of η and τ_{Q}. For parameter ranges outside the conditions determined in Supplementary Section 2, CS becomes low or NaN. This is because the synaptic weights were not updated, and their mean remained zero, so the structure of the synaptic weight space did not appear in the output space. Furthermore, when all elements of the distance $\mathbf{D}_{\text{w}}(t)$ between synaptic weights become zero–that is, when the synaptic weight values were never updated from their initial values–CS becomes NaN.

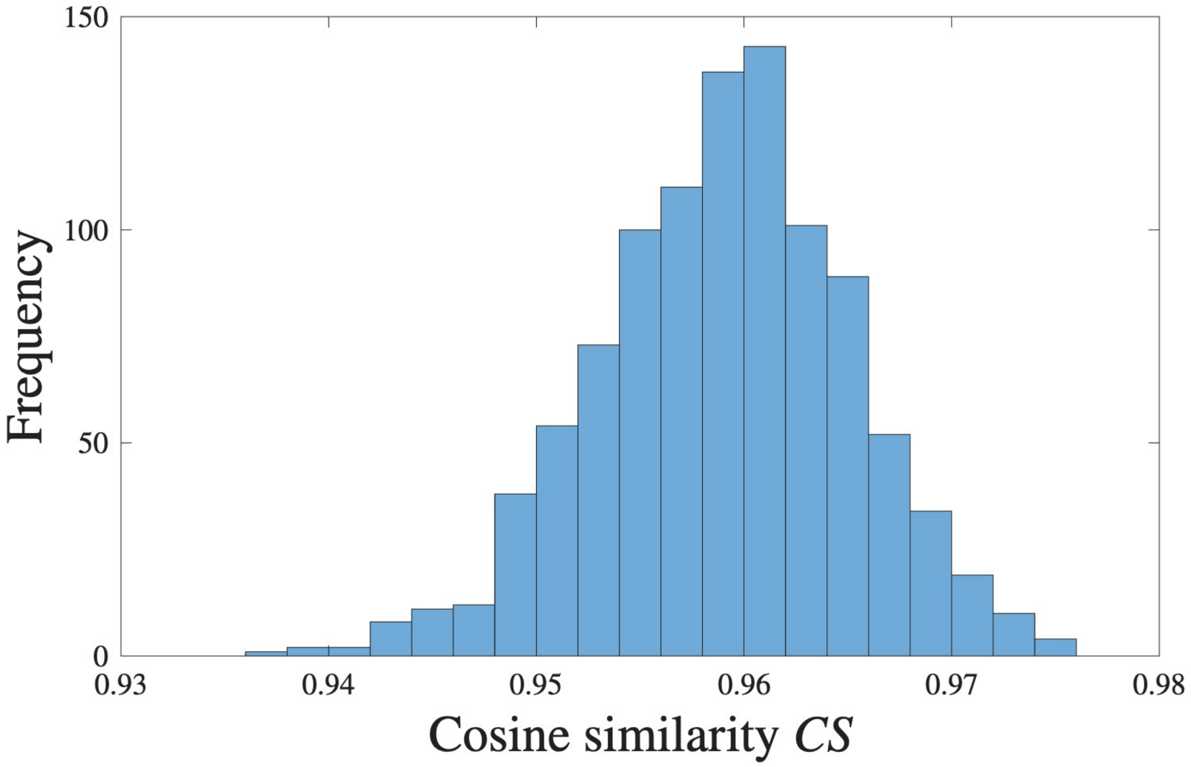

Figure S2. Histogram of the cosine similarity between the synaptic weights and outputs of the network in the distance space. The vertical and horizontal axes represent frequency and cosine similarity, respectively. The cosine similarity was obtained by changing the initial values of the synaptic weights of the network 1000 times. The frequency distribution approaches a normal distribution, as predicted by the central limit theorem.

4 HISTOGRAMS AND TWO-DIMENSIONAL DISTANCE MAPS

Figure S4 shows several histograms and two-dimensional distance maps of the synaptic weights, internal states, and outputs of the network. In Figures S4(**A**) to S4(**C**), the right, middle, and left histograms represent the frequency of the Euclidean distance between the synaptic weight matrices, the Euclidean distance between the internal state vectors, and the Hamming distance between the output vectors, respectively. In the middle histograms, when the synaptic weight values of the network are fixed to $\mathbf{W}^l(T+1)$, the internal state vector is obtained as follows:

$$\mathbf{P}^l(T+1) = [p_1^l(T+1), \cdots, p_i^l(T+1), \cdots, p_N^l(T+1)]^\top. \tag{S18}$$

For simplicity, the length of the spatiotemporal pattern is set to $T = 3$ in this experiment. Figures S4(**A**) to (**C**) show the learning results when the initial values of the synaptic weights are different. In the histograms, the colors represent the temporal order of spatial vectors. Yellow, green, light-blue, and blue represent cases where the first spatial vector is different ($k_1^l \neq k_1^{l'}$), the first spatial vector is the same ($k_1^l = k_1^{l'}$, $k_2^l \neq k_2^{l'}$), the first two spatial vectors are the same ($k_1^l = k_1^{l'}$, $k_2^l = k_2^{l'}$, $k_3^l \neq k_3^{l'}$), and the input spatiotemporal patterns are the same ($k_1^l = k_1^{l'}$, $k_2^l = k_2^{l'}$, $k_3^l = k_3^{l'}$), respectively. In Figure S4(**A**), peaks of the histograms in the synaptic weight, internal state, and output space are separated for each color. In this case, an order-nested structure can be confirmed in the output space. However, in Figures S4(**B**) and

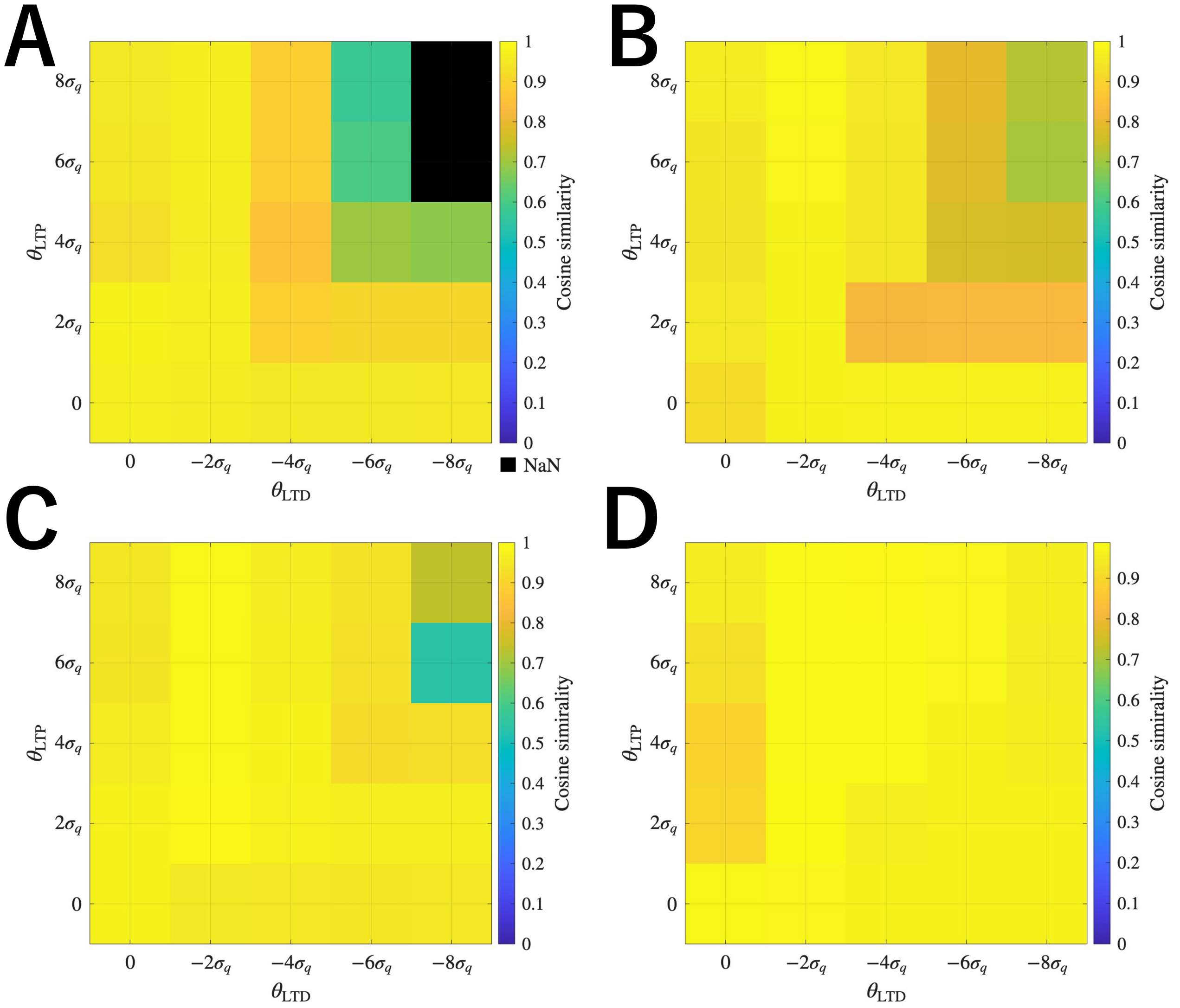

Figure S3. Average cosine similarity for various parameter values over 100 trials with different initial synaptic weights. $\theta_{\rm LTP}$, $\theta_{\rm LTD}$, $\tau_{\rm Q}$, and η are varied from 0 to $8\sigma_q$, from 0 to $-8\sigma_q$, with or without time history $\{0,\ 2.23\}$, and with small or large learning coefficients $\{0.5,\ 2\}$, respectively. **(A)** $\tau_{\rm Q} = 0$ and $\eta = 0.5$. **(B)** $\tau_{\rm Q} = 0$ and $\eta = 2$. **(C)** $\tau_{\rm Q} = 2.23$ and $\eta = 0.5$. **(D)** $\tau_{\rm Q} = 2.23$ and $\eta = 2$. The color bars represent cosine similarity. In the color bar of **(A)**, NaN indicates cases where cosine similarity cannot be calculated. The results show that high cosine similarity is robustly achieved when the parameter values are within the necessary conditions for learning derived in Supplementary Section 2, confirming the stability of the relationship.

S4(**C**), the peaks of the histograms overlap where indicated by red arrows. In these cases, the order-nested structure cannot be confirmed in the output space. In Figure S4(**B**), information reduction occurred due to dimensional compression caused by mapping from the synaptic weight space to the internal state space. Additionally, in Figure S4(**C**), the firing threshold of the neurons has not been adjusted appropriately. From the results, the order-nested structure formed in the synaptic weight space does not always appear in the output space. However, conversely, if the order-nested structure is confirmed in the output space, the fractal-like structure is also formed in the synaptic weight space.

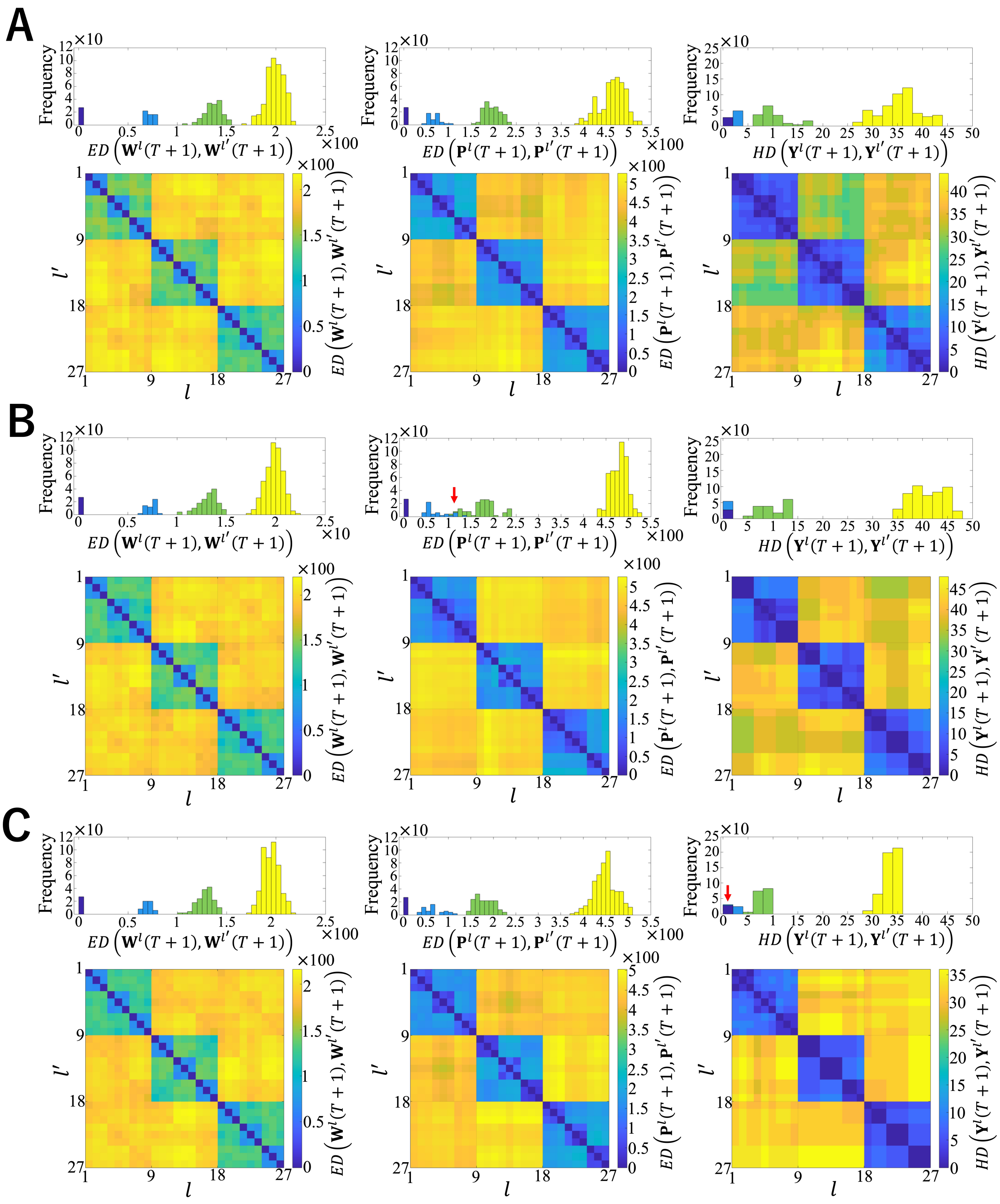

Figure S4. Histograms and two-dimensional distance maps of the synaptic weights, the internal states, and the outputs in the distance space. **(A)**, **(B)**, and **(C)** represent a different simulation result. Within each row, the left column shows the results for the synaptic weight space, the middle column for the internal state space, and the right column for the output space. The plots show both the distance map and a histogram of the distances. **(A)** A successful case where the fractal-like structure is clearly visible in all three spaces. **(B)** A case where the structure is lost in the internal state space (indicated by the red arrow showing overlapping peaks in the histogram) due to information compression. **(C)** A case where the structure is lost in the output space due to an improperly adjusted firing threshold. This analysis supports the conclusion that the output structure reflects the weight structure only under specific conditions.

5 ESTIMATING THE COMPRESSION DIMENSION IN MDS

Estimating a suitable compression dimension is necessary to project the dimension of synaptic weight space into a lower-dimensional distance space using the MDS method.

Figure S5 shows the eigenvalues of the symmetric matrix $\mathbf{G}_{\mathrm{w}}(t)\mathbf{G}_{\mathrm{w}}^{\top}(t)$ and ρ_n with respect to the compression dimension n. The asterisks in the figure represent the average eigenvalues when the initial synaptic weight values were changed 1000 times. The light blue color represents the maximum and minimum values of the 1000 trials. The red line in the figure shows the average of ρ_n when the initial value of the synaptic weight was changed 1000 times. The cumulative contribution ratio ρ_n is 0.47 and 0.55 when the compression dimension n is two and three, respectively. Additionally, we confirm that the eigenvalue decreases significantly after the third dimension. From these results, we conclude that a suitable compression dimension is two ($n = 2$).

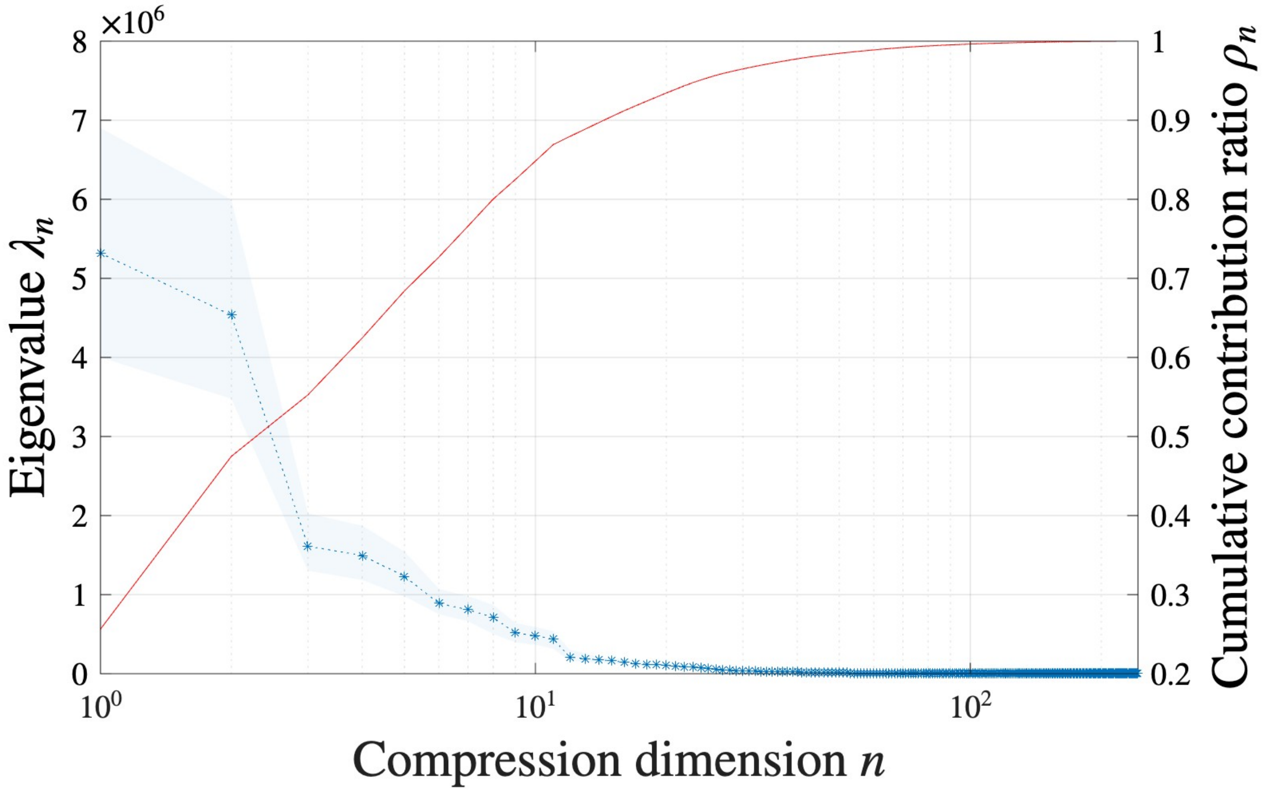

Figure S5. Eigenvalue (left scale) and cumulative contribution ratio (right scale) with respect to compression dimension. Asterisks represent the average eigenvalues. The light-blue area represents the maximum and minimum values of the eigenvalues. The red line represents the average of ρ_n.

6 COMPARISON WITH OTHER DIMENSION REDUCTION METHODS

Various dimension reduction methods have been proposed to map information from high-dimensional spaces to low-dimensional spaces while preserving as much information as possible. Among these, Isomap (Tenenbaum et al., 2000) and UMAP (McInnes et al., 2020) methods are often used. The Isomap method calculates the shortest path between each point in the dataset and maps them to a lower dimension

while preserving the distance. It calculates the Euclidean distance on the manifold using the k-nearest neighbors method to represent the geodesic distance between datasets in a lower dimension using the MDS method. Therefore, the Isomap method yields nearly identical results to the MDS method. The UMAP method also utilizes the k-nearest neighbors approach and assigns weights to each dataset. It employs a force-directed graph drawing algorithm to generate a low-dimensional representation. This algorithm calculates attractive and repulsive forces between each vertex generated from the dataset, moves the vertices while decreasing the temperature parameter, and determines the optimal vertex positions. Unlike the Isomap and MDS methods, it represents the relative relationships between vertices as an energy minimization problem, allowing for the simultaneous analysis of both the local and global structures inherent in the dataset.

Both the Isomap and UMAP methods create graphs based on k_{NN}-nearest neighbors. These methods can adjust k_{NN} to analyze the local and global structures of the dataset. We can analyze the local and global structure of the dataset when k_{NN} is small and large, respectively. In other words, k_{NN} is set to a small value if we want to observe a fine structure, while k_{NN} is set to a large value if we want to observe an overall structure.

Figure S6 shows the analysis results obtained by varying k_{NN} from 100 to 200 using the Isomap method. When $k_{\mathrm{NN}} < K^{T-1} = 81$, the number of plots in the low-dimensional distance space falls below $K^T = 243$. Therefore, the minimum k_{NN} is set to 100. The horizontal and vertical axes in the figure represent Euclidean distances, respectively. The Isomap method compresses dimensions based on distances calculated using the k_{NN}-nearest neighbors approach and then visualizes the data using the MDS method. Therefore, results analyzed using the Isomap method with a large k_{NN} and the MDS method are equivalent. Comparing the results in Figure S6(**D**) and Figure 4 confirms that almost the same results were obtained. On the other hand, when k_{NN} is small, we can analyze the local structure. However, Figure S6(**A**) doesn't show the local self-similar structure. Figure S6

Figure S7 shows the analysis results obtained by varying k_{NN} from 100 to 200 using the UMAP method. The horizontal and vertical axes in the figure represent Euclidean distances, respectively. Figure S7(**A**) shows local self-similar structures, but lacks the global self-similarity seen in Figure S7(**D**) (where the distances between the centers of each cluster are equal, and the distances between the centers of each small cluster are also equal). On the other hand, Figure S7(**D**) shows a global self-similar structure, but it lacks the local self-similar structure seen in Figure S7(**A**) (where the three main clusters each split into three smaller clusters, and those three smaller clusters further divided into even smaller clusters).

However, neither method allows for the simultaneous observation of fine- and global-scale structures by adjusting k_{NN}. Therefore, to visualize the fine- and global-scale structures as shown in Figure 5, the method we propose—combining MDS with adjustment of cluster center coordinates—is necessary.

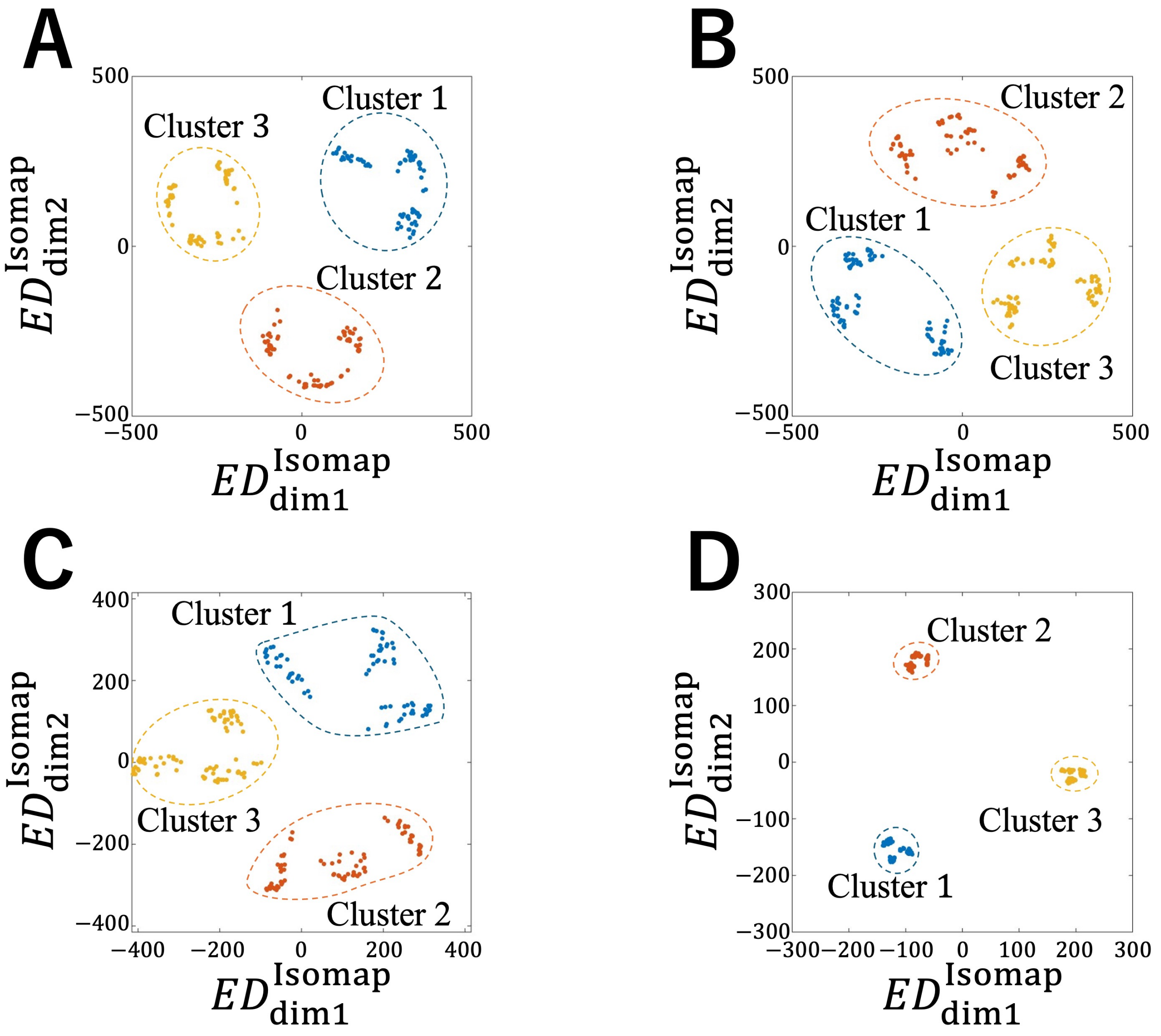

Figure S6. Position of synaptic weights represented in a low-dimensional distance space by the Isomap method. **(A)** $k_{\text{NN}} = 100$. **(B)** $k_{\text{NN}} = 120$. **(C)** $k_{\text{NN}} = 140$. **(D)** $k_{\text{NN}} = 200$.

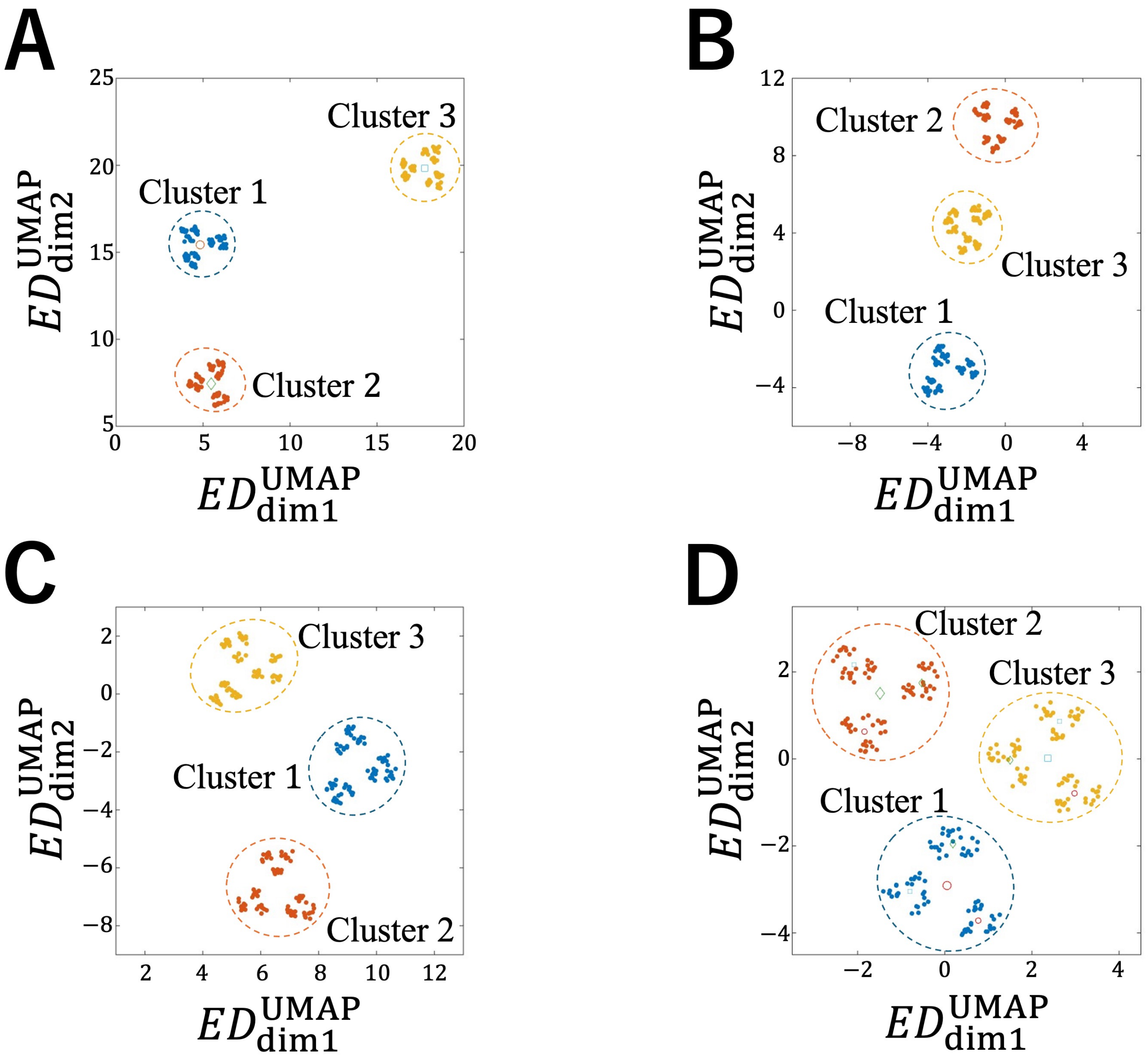

Figure S7. Position of synaptic weights represented in a low-dimensional distance space by the UMAP method. **(A)** $k_{\mathrm{NN}} = 100$. **(B)** $k_{\mathrm{NN}} = 120$. **(C)** $k_{\mathrm{NN}} = 140$. **(D)** $k_{\mathrm{NN}} = 200$.

7 FRACTAL STRUCTURE WITH NOISY INPUTS

θ_{LTP} and θ_{LTD} are parameters that regulate pattern integration and separation, respectively. When θ_{LTP} is small, pattern integration dominates; when θ_{LTD} is small (approaching 0), pattern separation dominates. Numerical experiments were conducted using the parameters described in Section 3, with white noise added to the input. The results are shown in Figure S8. Figure S8 shows the results of analyzing the distance matrix between synaptic weights using the MDS method. The two horizontal axes represent the MDS projection (Euclidean distance), and the vertical axis represents discrete time. Figures S8(**A**), (**B**), (**C**), and (**D**) show the results with noise levels of $0.1\sigma_p$, $0.2\sigma_p$, $0.5\sigma_p$, and σ_p applied, respectively, based on the standard deviation (Equation S5) of the internal state of a neuron without noise. The results in Figure S8(**D**) show that due to an excellent separation capability of the STLR, patterns containing noise are significantly separated as distinct inputs. Consequently, at $t = 5$, the distances between synaptic weights are evenly distributed. The MDS analysis results at $t = 5$ for Figures S8(**A**), (**B**), (**C**), and (**D**) are shown in Figures S9, S10, S11, and S12, respectively. In each figure, (**A**), (**B**), (**C**), and (**D**) show the MDS analysis results for the entire set at $t = 5$, the MDS analysis results within Cluster 1, the MDS analysis results within Cluster 2, and the MDS analysis results within Cluster 3, respectively. The results in Figures S9–S12 show that even though the cluster regions overlap at $t = 5$, the areas within each cluster do not overlap. In other words, fractal coding through the STLR is achieved, but due to noise effects, only the initially input pattern is not encoded correctly. This result suggests that the network exhibits dominant separation capability because the value of θ_{LTP} is greater than the absolute value of θ_{LTD}. Therefore, Figure S13 shows the results when the noise of σ_p magnitude was applied after changing θ_{LTP} from 4 to 2. Figure S13(**A**) shows that at $t = 5$, the distances between synaptic weights, which were uniformly distributed, are separated according to the initial input. Furthermore, Figure S13(**B**) shows the MDS analysis of the entire data at $t = 5$. Compared to Figure S12(**A**), clusters corresponding to the initial input patterns are separated. This result suggests that in a high-noise environment, decreasing the θ_{LTP} value can make pattern integration more dominant.

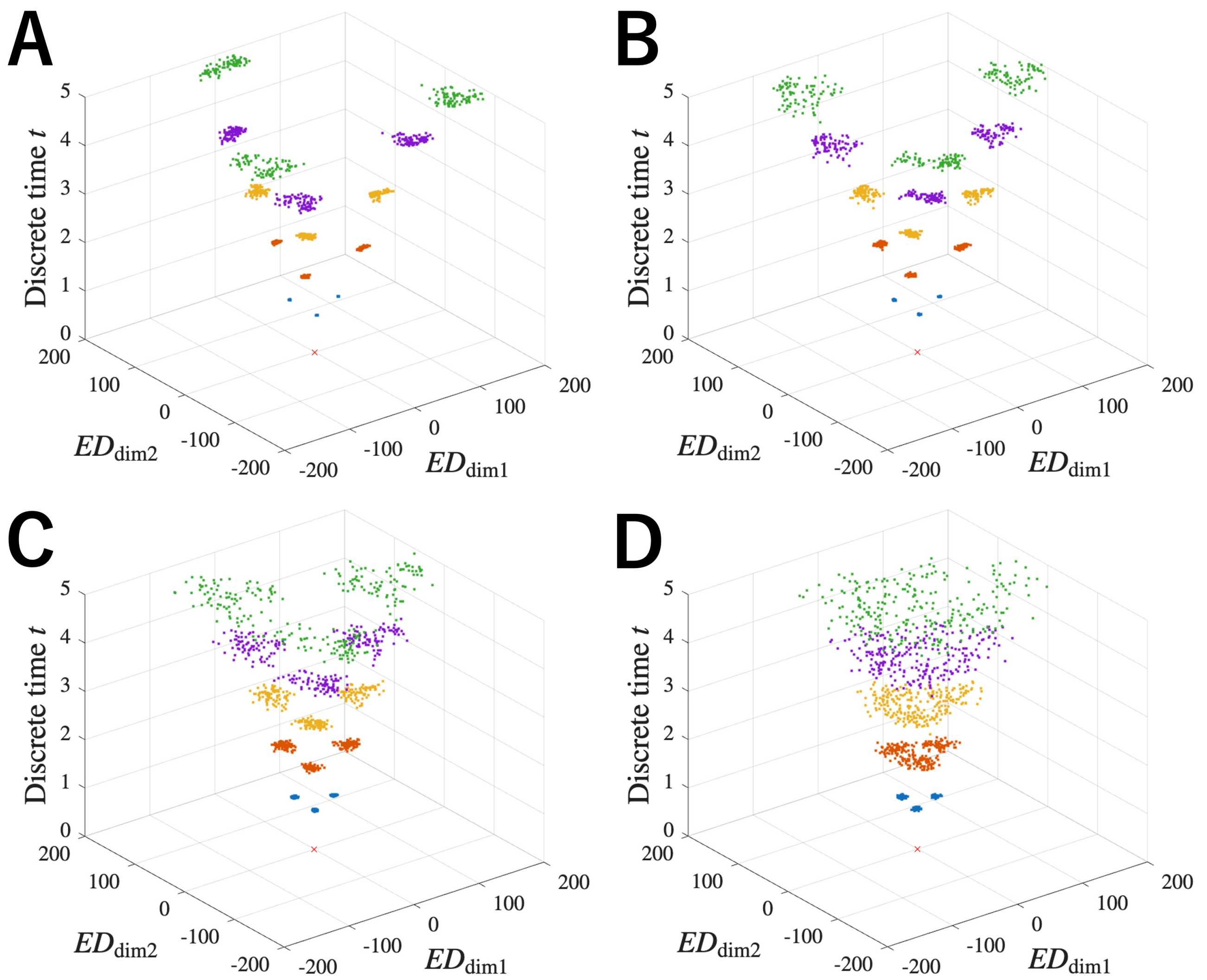

Figure S8. Time evolution of the synaptic weight structure in the distance space under varying amplitude of input noise. **(A)** noise $0.1\sigma_p$. **(B)** $0.2\sigma_p$. **(C)** $0.5\sigma_p$. **(D)** σ_p. As the noise amplitude increases, the clarity of the branching structure degrades, and the final clusters at $t = 5$ become more diffuse and begin to overlap.

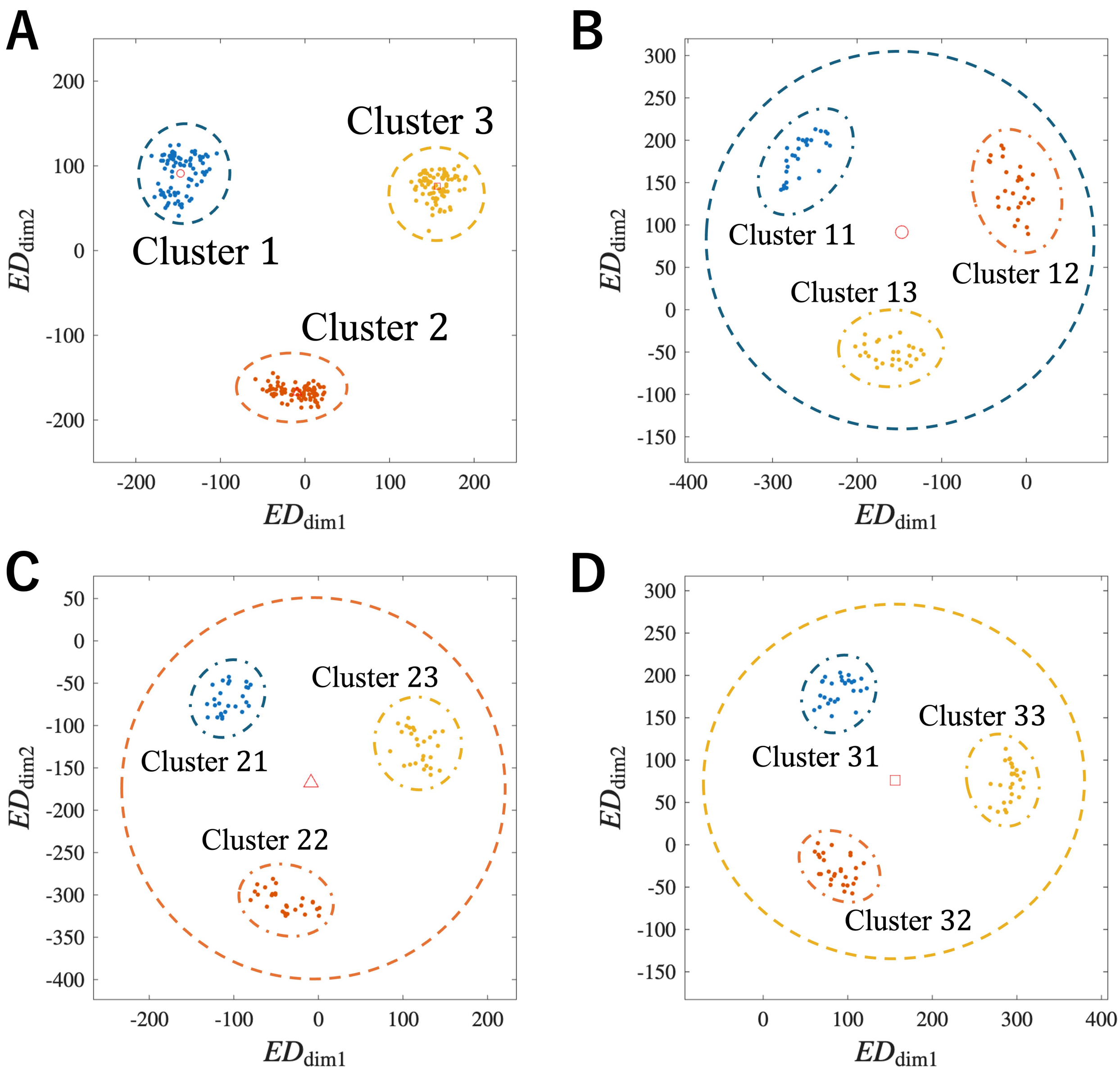

Figure S9. Recursive MDS analysis of the final state ($t = 5$) under varying noise amplitude $0.1\sigma_p$. **(A)** overall. **(B)** in Cluster 1. **(C)** in Cluster 2. **(D)** in Cluster 3.

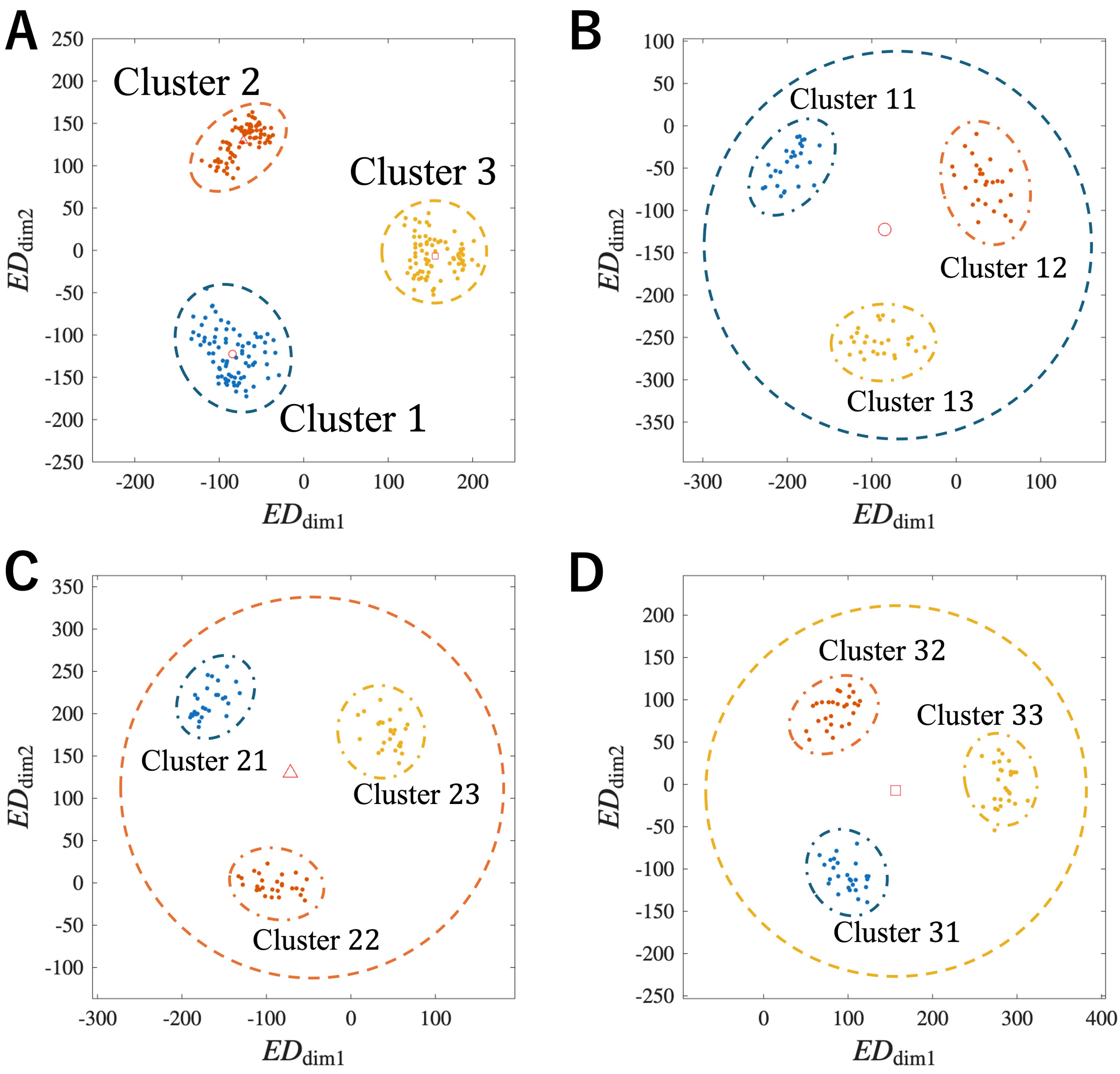

Figure S10. Recursive MDS analysis of the final state ($t = 5$) under varying noise amplitude $0.2\sigma_p$. **(A)** overall. **(B)** in Cluster 1. **(C)** in Cluster 2. **(D)** in Cluster 3.

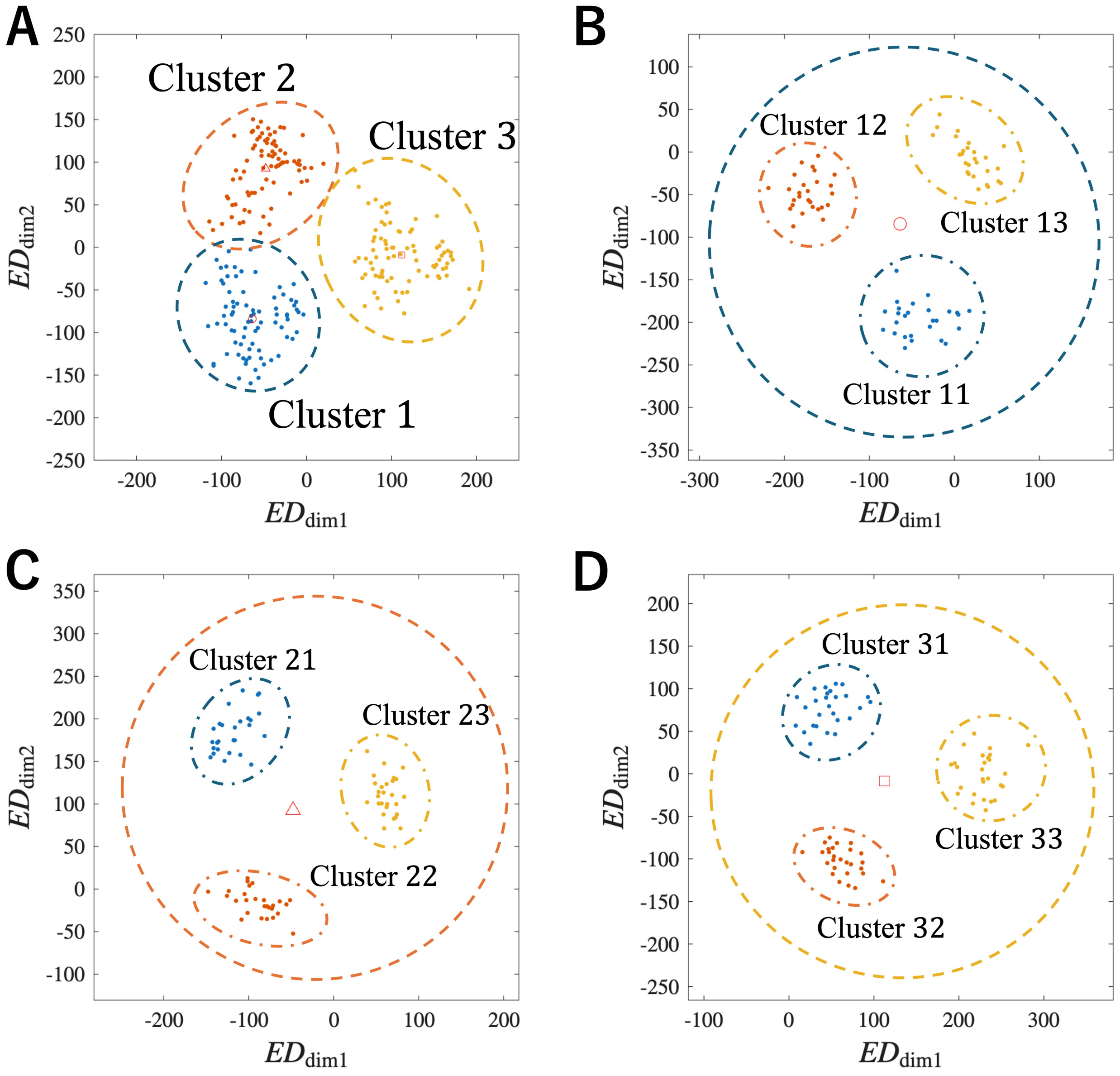

Figure S11. Recursive MDS analysis of the final state ($t = 5$) under varying noise amplitude $0.5\sigma_p$. **(A)** overall. **(B)** in Cluster 1. **(C)** in Cluster 2. **(D)** in Cluster 3.

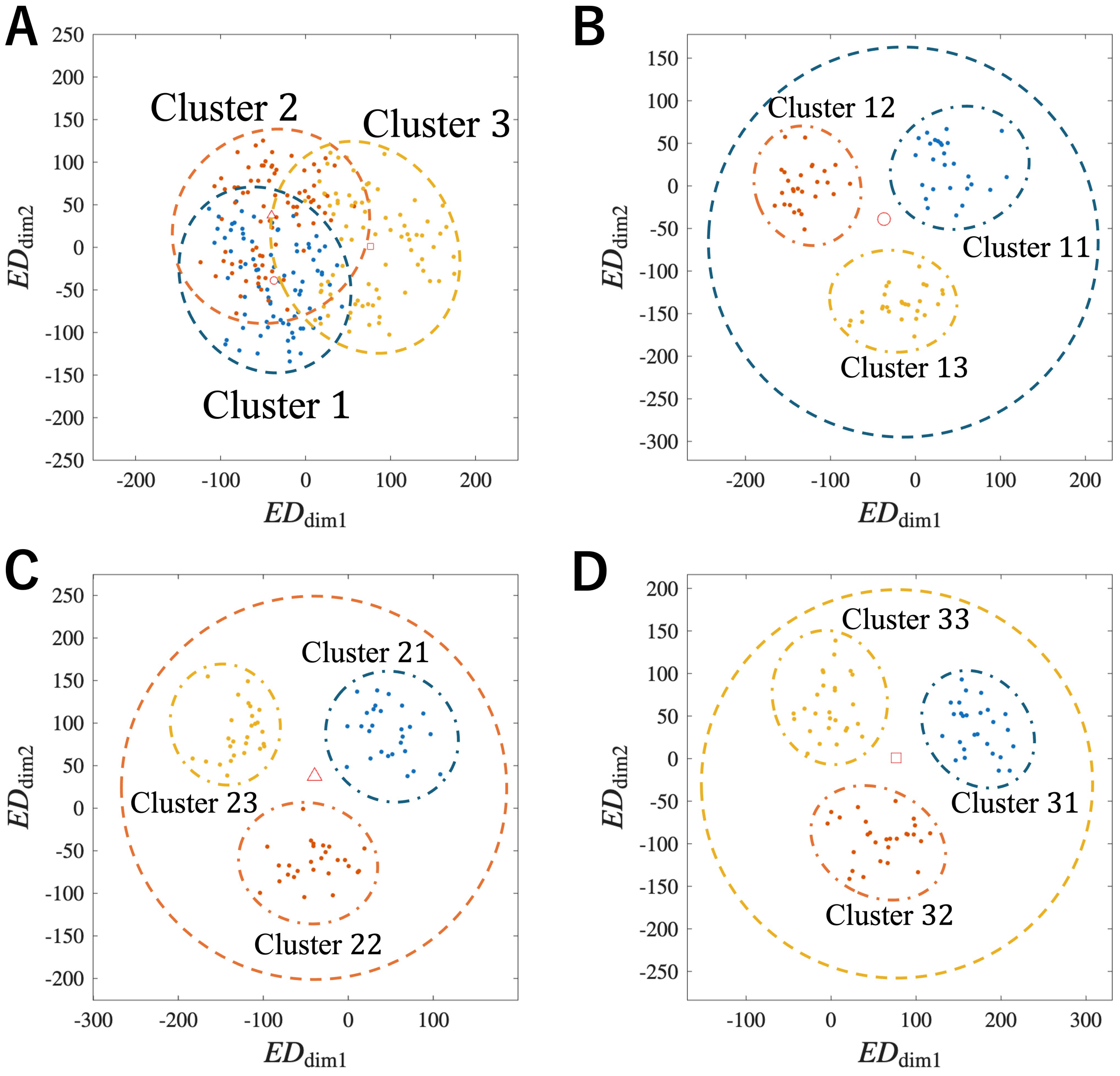

Figure S12. Recursive MDS analysis of the final state ($t = 5$) under varying noise amplitude σ_p. **(A)** overall. **(B)** in Cluster 1. **(C)** in Cluster 2. **(D)** in Cluster 3.

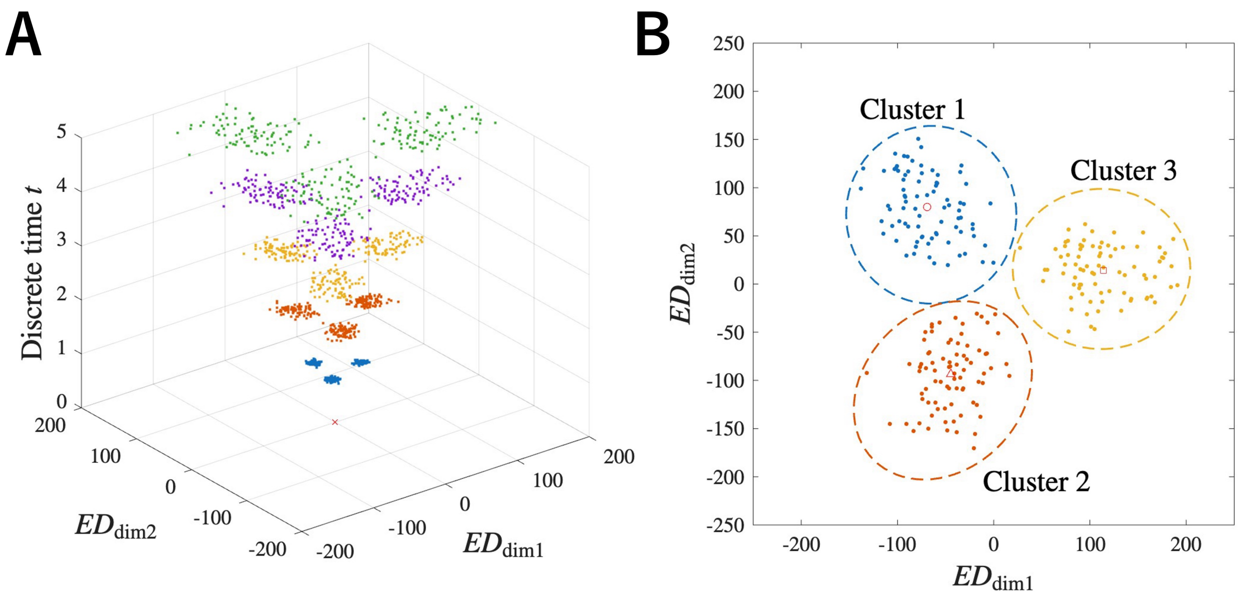

Figure S13. Effect of lowering the LTP threshold in a high-noise environment. MDS analysis with noise σ_p by changing θ_{LTP} from 4 to 2. **(A)** the time evolution. **(B)** overall at $t = 5$. This result indicates that lowering the LTP threshold enhances the input spatial pattern completion ability, allowing it to effectively counteract the noise and restore accurate spatiotemporal pattern separation.

8 ESTIMATION OF FRACTAL DIMENSION USING THE BOX-COUNTING METHOD

The fractal dimension is estimated using the box-counting method. The results of applying the box-counting method to $\mathbf{G}_{\mathrm{w}}^{\mathrm{adj}}(t)$ are shown in Figure S14. The horizontal and vertical axes in Figure S14 represent the natural logarithms of the box size and number of boxes containing points, respectively.

The box-counting method estimates the fractal dimension using the following procedure. First, Figure 5 is divided into boxes of size δ, and the number of boxes $B(\delta)$ that contain points is counted. The procedure is then repeated by increasing δ and counting $B(\delta)$ again. The number of boxes containing points is plotted against box size, and linear regression is used to approximate the line. The absolute value of the slope of the line corresponds to the fractal dimension. Consequently, the fractal dimension shown in Figure 5 is estimated to be 1.415.

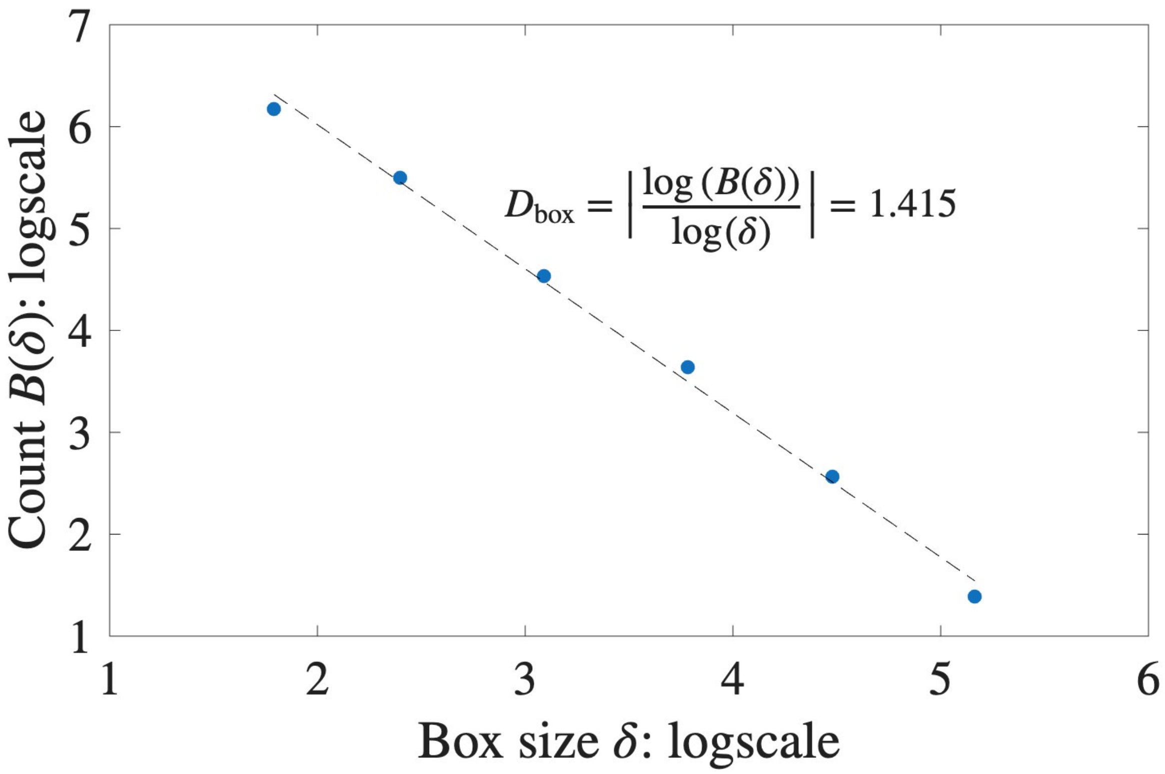

Figure S14. Number of boxes containing points within the box relative to box size. Blue circles indicate the results of measurements using the box-counting method in Figure 5. The dashed line represents the results of linear regression.

9 COMPARISON WITH OTHER ESTIMATION METHODS OF FRACTAL DIMENSION

The box-counting method divides an image into boxes of arbitrary size to measure the self-similarity. Similarly, methods exist to measure lacunarity (Mandelbrot, 1983) in an image or to measure local fractal dimension by dividing the image into some ranges of arbitrary size.

The lacunarity method is similar to the box-counting method (Smith et al., 1996). The box-counting method estimates fractal dimension based on the relationship between box size and the number of points contained within the divided boxes. The lacunarity method, on the other hand, counts the number of points contained within a box and calculates the coefficient of variation as shown in Figure S15. This method estimates the lacunarity by averaging the coefficient of variation. Thus, the lacunarity shown in Figure 5 is estimated to be 0.6048. If the coefficient of variation is constant with respect to box size, it indicates the texture of an image is uniform. The results in Figure S15 quantitatively confirm that the texture in Figure 5 is not uniform. Furthermore, a negative correlation exists between the lacunarity and the fractal dimension (Smith et al., 1996), allowing the fractal dimension of the target image to be estimated as well.

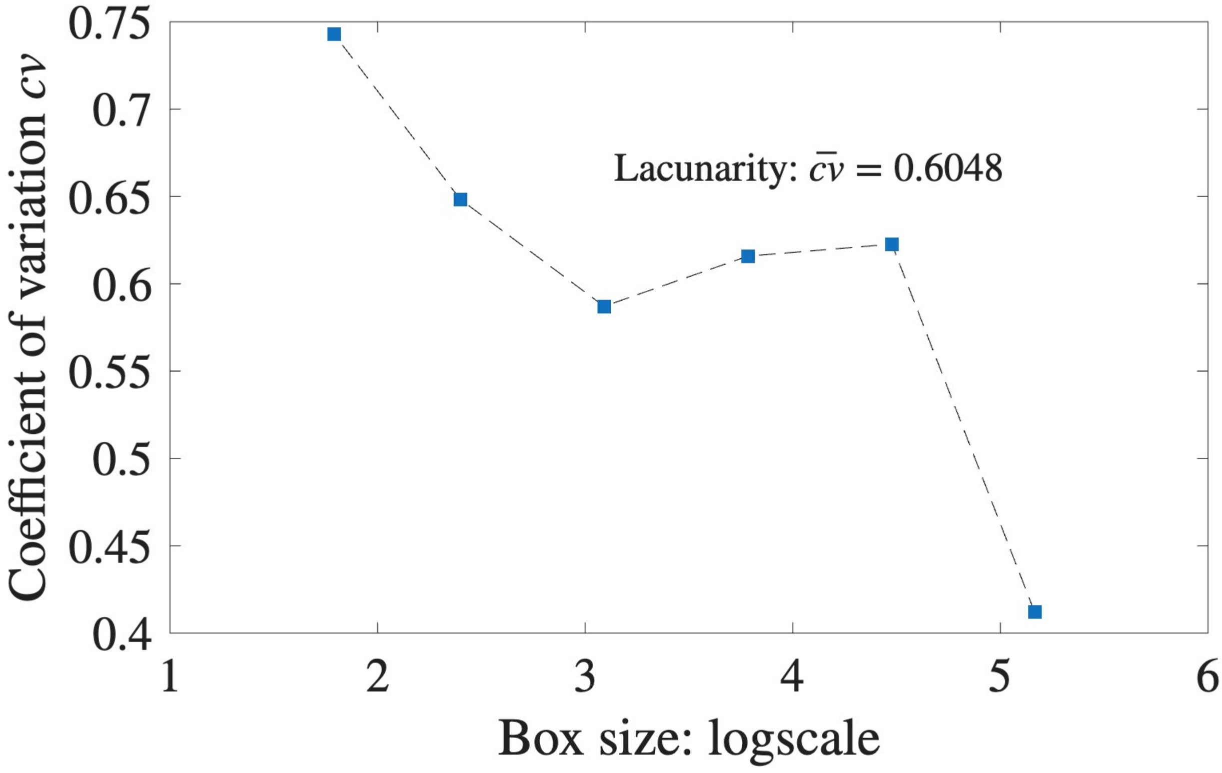

Figure S15. Coefficient of variation with respect to box size. The coefficient of variation is calculated by setting boxes to arbitrary sizes and counting the points within each box in the image. The coefficient of variation is calculated for the count obtained for each box. Blue squares indicate the results of measurements using the lacunarity method in Figure 5. If an image's texture is uniform, the coefficient of variation with respect to box size is constant. $\bar{cv}$ is the average of the coefficient of variation cv and indicates the lacunarity.

The local fractal dimension can be estimated using the mass-radius method (Landini and Rippin, 1993). The mass-radius method involves placing a box of varying sizes at any point in the image and counting the number of points contained within that box. The local fractal dimension is derived from the relationship between the box size and the count. This method is similar to the box-counting method, but as shown in Figure S16, the local fractal dimension is larger than that estimated by the box-counting method. The mass-radius method estimates the average of the local fractal dimensions at various positions within an

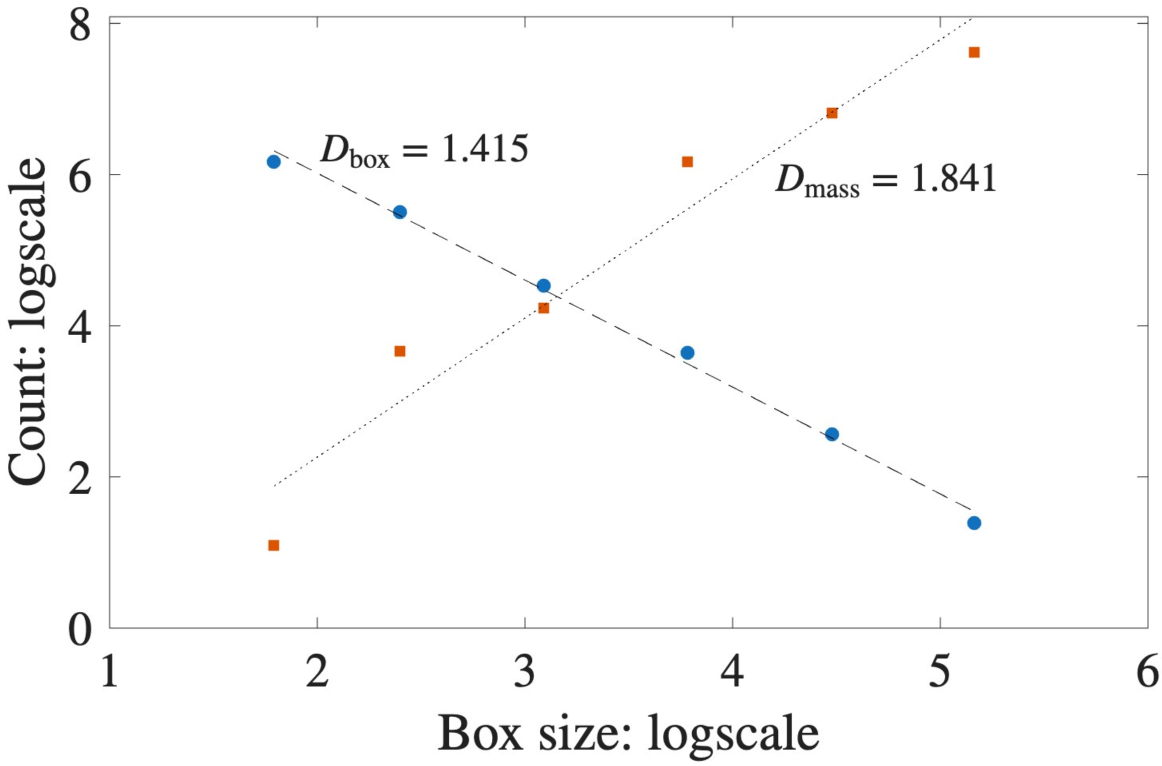

Figure S16. Characteristics of counts with respect to box size obtained by the box-counting method and the mass-radius method. Blue circles (from the results in Figure S14) and orange squares indicate the results of measurements using the box-counting and lacunarity methods in Figure 5. The dashed and dotted lines represent the results of linear regression for blue circles and orange squares, respectively. D_{box} and D_{mass} are the estimated fractal dimensions by the box-counting method and the mass-radius method, respectively.

image. Consequently, multiple local fractal dimensions are obtained. Figure S17 shows the distribution of these fractal dimensions. Based on the characteristics of this distribution, it not only estimates the fractal dimension of the target image but also investigates the structural features of the image. Furthermore, by calculating the average of the local fractal dimensions, the fractal dimension of the target image can be estimated.

Figure S18, S19, and S20 shows the fractal dimension, the lacunarity, and the average of the local fractal dimension obtained by the box-counting method, the lacunarity method, and the mass-radius method, respectively, when the parameters θ_{LTP}, θ_{LTD}, τ_{Q}, and η are varied from 0 to $8\sigma_q$, from 0 to $-8\sigma_q$, with or without time history $\{0,\ 2.23\}$, and with small or large learning coefficients $\{0.5,\ 2\}$, respectively. The fractal dimension, the lacunarity, and the average of the local fractal dimension are the average values obtained by changing the initial synaptic weights 100 times.

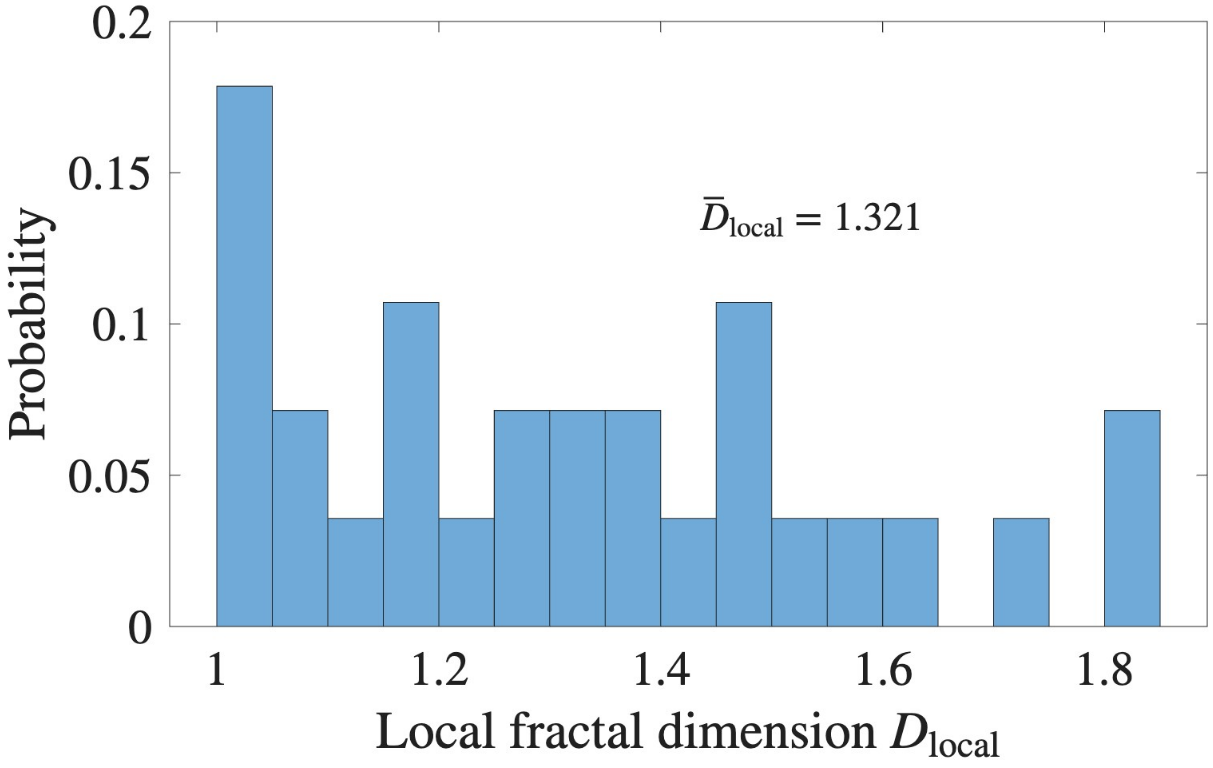

Figure S17. Distribution of local fractal dimension. The horizontal and vertical axes represent the local fractal dimension and probability, respectively. The histogram results show the estimated local fractal dimensions and their corresponding probabilities at various positions, as shown in Figure 5, obtained using the mass-radius method. $\bar{D}_{\mathrm{local}}$ is the average of the local fractal dimensions.

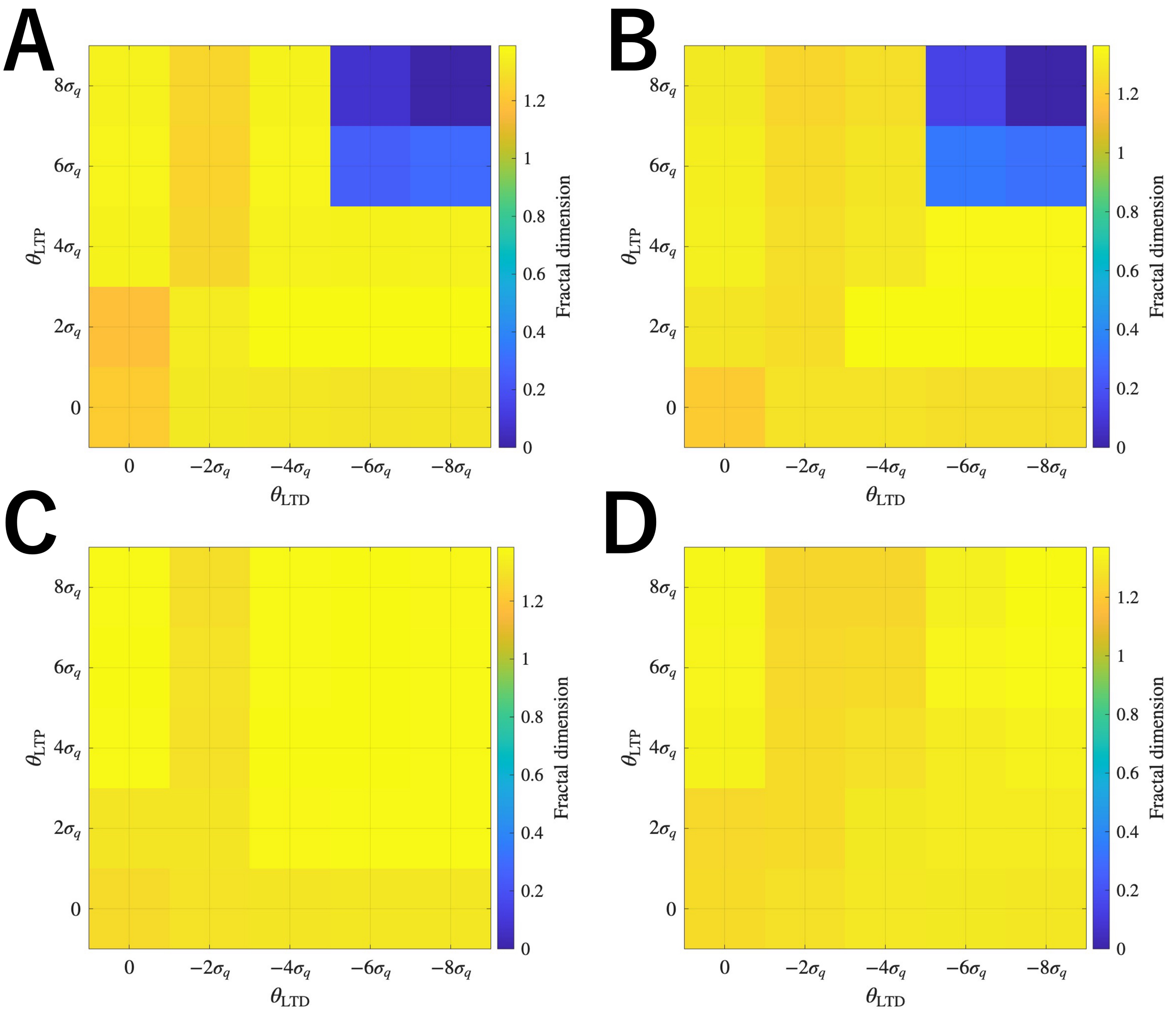

Figure S18. Parameter sensitivity of the estimated fractal dimension by the box-counting method. The vertical axis represents the LTP threshold, and the horizontal axis represents the LTD threshold, respectively. Each panel corresponds to different settings for the time constant $\tau_{\rm Q}$ and learning rate η. The color in the maps represents the calculated fractal dimension. **(A)** $\tau_{\rm Q} = 0$ and $\eta = 0.5$. **(B)** $\tau_{\rm Q} = 0$ and $\eta = 2$. **(C)** $\tau_{\rm Q} = 2.23$ and $\eta = 0.5$. **(D)** $\tau_{\rm Q} = 2.23$ and $\eta = 2$. These results demonstrate that a non-trivial fractal dimension (i.e., a value greater than 0, indicated by the blue region) emerges robustly within a specific parameter regime that aligns with the necessary conditions for learning.

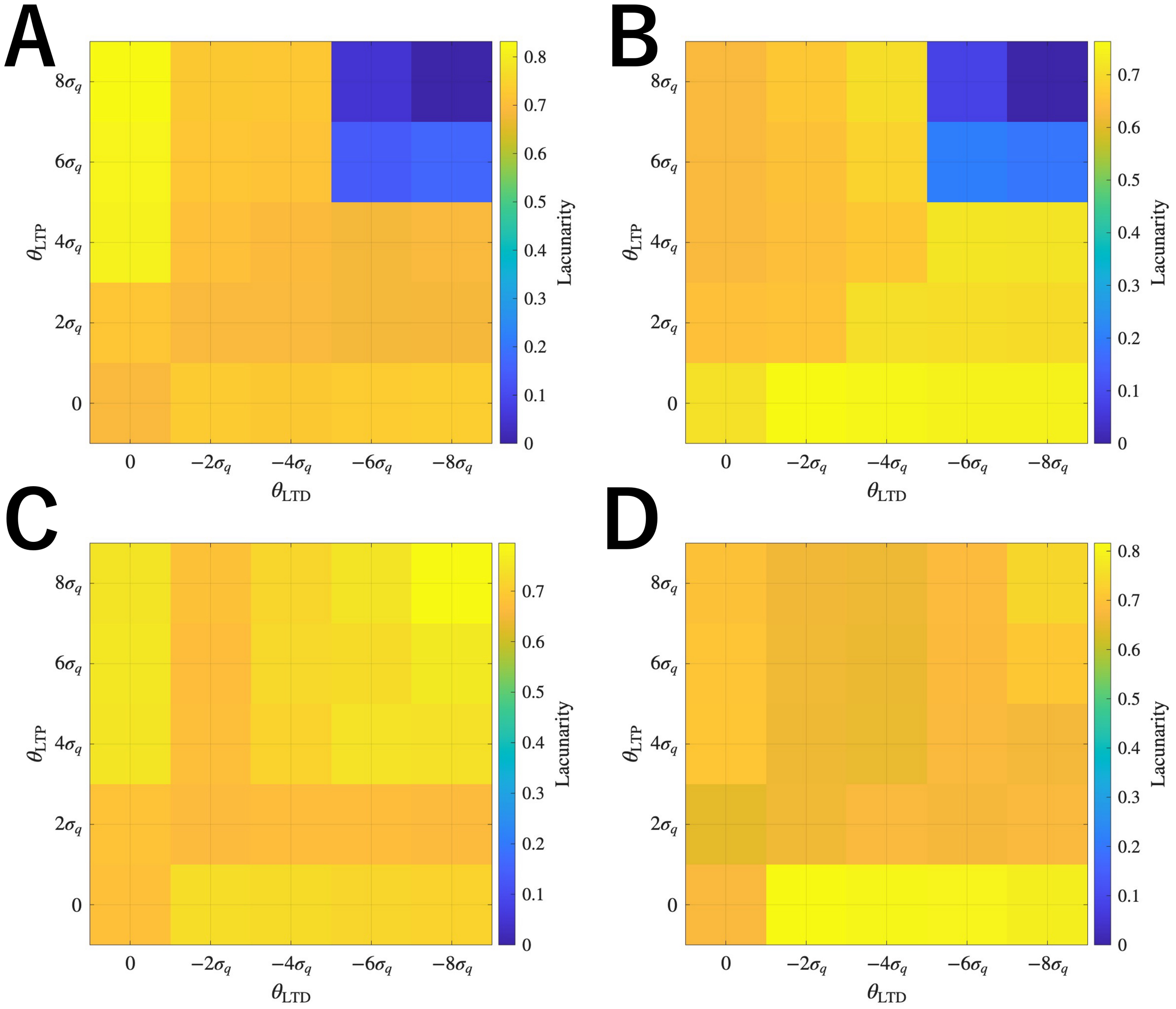

Figure S19. Parameter sensitivity of the estimated lacunarity by the lacunarity method. The vertical axis represents the LTP threshold, and the horizontal axis represents the LTD threshold, respectively. Each panel corresponds to different settings for the time constant τ_{Q} and learning rate η. The color in the maps represents the calculated lacunarity. **(A)** $\tau_{\text{Q}} = 0$ and $\eta = 0.5$. **(B)** $\tau_{\text{Q}} = 0$ and $\eta = 2$. **(C)** $\tau_{\text{Q}} = 2.23$ and $\eta = 0.5$. **(D)** $\tau_{\text{Q}} = 2.23$ and $\eta = 2$. These results demonstrate that a non-trivial lacunarity (i.e., a value greater than 0, indicated by the blue region) emerges robustly within a specific parameter regime that aligns with the necessary conditions for learning.

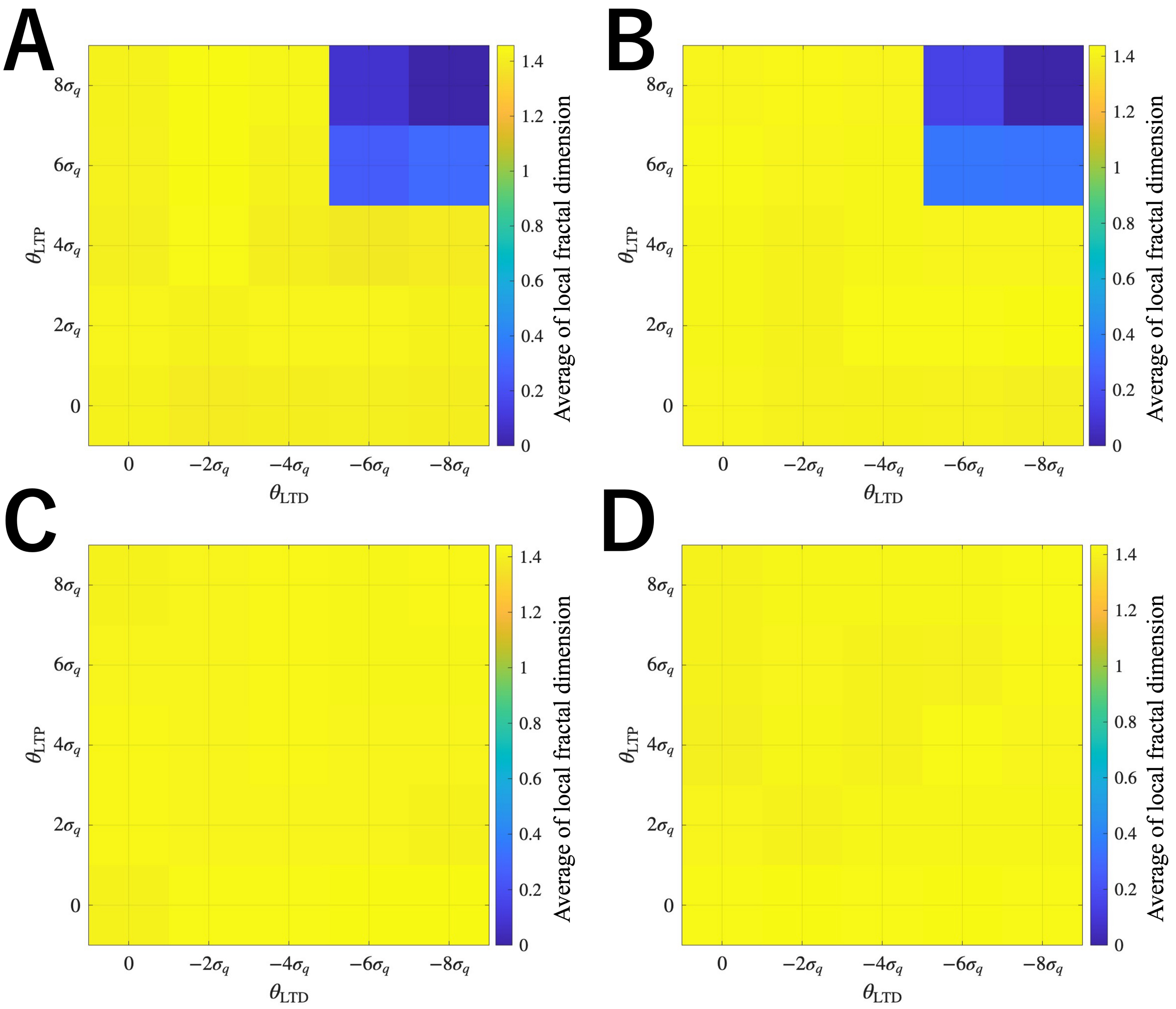

Figure S20. Parameter sensitivity of the average of local fractal dimensions, by the mass-radius method. The vertical axis represents the LTP threshold, and the horizontal axis represents the LTD threshold, respectively. Each panel corresponds to different settings for the time constant $\tau_{\rm Q}$ and learning rate η. The color in the maps represents the calculated fractal dimension. **(A)** $\tau_{\rm Q} = 0$ and $\eta = 0.5$. **(B)** $\tau_{\rm Q} = 0$ and $\eta = 2$. **(C)** $\tau_{\rm Q} = 2.23$ and $\eta = 0.5$. **(D)** $\tau_{\rm Q} = 2.23$ and $\eta = 2$. These results demonstrate that a non-trivial fractal dimension (i.e., a value greater than 0, indicated by the blue region) emerges robustly within a specific parameter regime that aligns with the necessary conditions for learning.

10 INPUT-DRIVEN EXPANSION SYSTEM

A feedforward neural network with STLR synapses can be represented as an input-driven expansion system. Therefore, we describe the input-driven expansion system as an iterative function system (IFS) to analyze the mathematical formation of self-similar structures. In an IFS, contraction maps and expansion maps are opposed concepts, although they actually represent the same mathematical object viewed from different perspectives. Understanding this duality is key to linking the IFS with chaotic dynamics. The repeller of a piecewise expansion map F can be constructed as an attractor in the IFS. This IFS is formed by the collection of F's inverse branches. If F is an expansion map, its local inverse must necessarily be a contraction map (Simon et al., 2001; Rempe-Gillen and Urbański, 2015). This fundamental relationship unifies the two domains of stable geometric structures (attractors) and unstable chaotic dynamics (repellers).

The input-driven expansion system is described as an IFS on a distance space, and the mechanism by which it forms the self-similar structure characteristic of fractals is analyzed mathematically.

We describe an IFS that operates on $\mathbf{g}(t)$, the two-dimensional representation of the synaptic weights $\mathbf{W}(t)$ obtained through the MDS method. The IFS is constructed such that the geometric relationships in the low-dimensional space $\mathbf{g}(t)$ reflect the distance in the high-dimensional synaptic weights space $\mathbf{W}(t)$.

$$\mathbf{W}(t) \in \mathbb{R}^{N\times M} \quad \overset{\text{MDS}}{\longrightarrow} \quad \mathbf{g}(t) \in \mathbb{R}^2, \tag{S19}$$

$$\mathbf{g}(t+1) \quad = \quad \bigcup_{k}^{K} F_k(\mathbf{g}(t)), \tag{S20}$$

$$F_k(\mathbf{g}(t)) \quad = \quad \mathbf{g}(t) + \gamma_k(t)[0 \;\; 1] \begin{bmatrix} \cos(\phi_k(t)) & \sin(\phi_k(t)) \\ -\sin(\phi_k(t)) & \cos(\phi_k(t)) \end{bmatrix}, \tag{S21}$$

$$\tag{S22}$$

where $\gamma_k(t)$ and $\phi_k(t)$ are an expansion coefficient and a phase angle based on $\mathbf{g}(t)$ at $k = 1$, $\phi_k(t) = 0$, respectively. Numerical simulations are performed to determine the expansion coefficient and phase angle. The results are shown in Figure S21. From these results, it is evident that the expansion coefficient is dependent on time. Therefore, we determine $\gamma_k(t+1) = 0.424\gamma_k(t)$ the average value of the results in Figure S21. Furthermore, the phase angles are $\phi_1(t) = 0$, $\phi_2(t) = 2.08 \approx 2\pi/3$, and $\phi = -2.12 \approx -2\pi/3$, respectively.

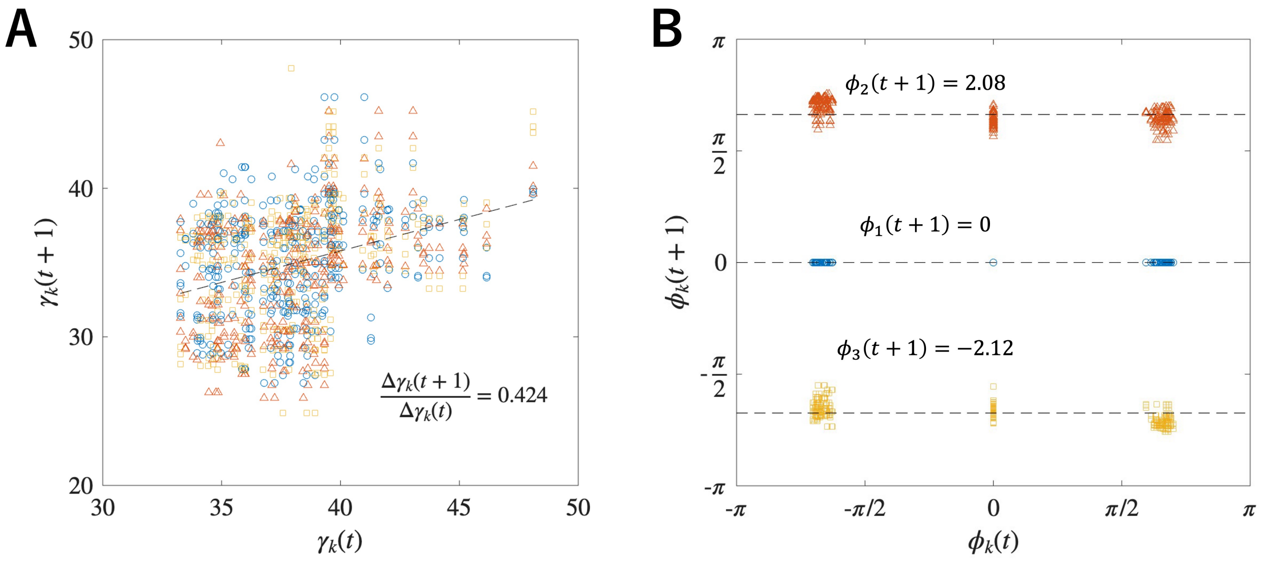

Figure S21. Return map. **(A)** Expansion coefficient return map. The horizontal and vertical axes represent the expansion coefficients at time t and $t + 1$, respectively. **(B)** Phase angle return map. The horizontal and vertical axes represent the phase angles at time t and $t + 1$, respectively.

REFERENCES

Feller, W. (1968). *An Introduction to Probability Theory and Its Applications*, vol. 1 (Wiley)

Landini, G. and Rippin, J. W. (1993). Notes on the implementation of the mass – radius method of fractal dimension estimation. *Bioinformatics* 9, 547–550. doi:10.1093/bioinformatics/9.5.547

Mandelbrot, B. B. (1983). *The fractal geometry of nature* (W. H. Freeman and Comp.)

[Dataset] McInnes, L., Healy, J., and Melville, J. (2020). Umap: Uniform manifold approximation and projection for dimension reduction

Rempe-Gillen, L. and Urbański, M. (2015). Non-autonomous conformal iterated function systems and moran-set constructions. *Transactions of the American Mathematical Society* 368, 1979–2017. doi:10.1090/tran/6490

Simon, K., Solomyak, B., and Urbański, M. (2001). Hausdorff dimension of limit sets for parabolic ifs with overlaps. *Pacific Journal of Mathematics* 201, 441–478. doi:10.2140/pjm.2001.201.441

Smith, T., Lange, G., and Marks, W. (1996). Fractal methods and results in cellular morphology — dimensions, lacunarity and multifractals. *Journal of Neuroscience Methods* 69, 123–136. doi:10.1016/S0165-0270(96)00080-5

Springer, M. D. (1979). *The Algebra of Random Variables*. Wiley series in probability and mathematicalstatistics (John Wiley & Sons)

Tenenbaum, J. B., de Silva, V., and Langford, J. C. (2000). A global geometric framework for nonlinear dimensionality reduction. *Science* 290, 2319–2323. doi:10.1126/science.290.5500.2319
